# Glycine: The missing link between carbohydrate and xenobiotic metabolism in the maturing human hepatocyte

**DOI:** 10.1016/j.isci.2026.116113

**Published:** 2026-05-28

**Authors:** Victoria Pozo Garcia, Tuğçe S. Çobanoğlu, Konstantina Riga, Suraj Sharma, Paul Jennings, J. Chris Vos, Sofia Moco

**Affiliations:** 1 Department of Chemistry and Pharmaceutical Sciences, Amsterdam Institute of Molecular and Life Sciences (AIMMS), Vrije Universiteit Amsterdam, De Boelelaan 1108, Amsterdam 1081 HZ, the Netherlands; 2 Department of Biomedicine, University of Bergen, Bergen 5020, Norway

**Keywords:** biological sciences, cell biology, metabolomics

## Abstract

Modulation of cellular metabolism is crucial in pluripotent stem cell (PSC) development and differentiation. Glycine was shown to promote liver cell maturation, boosting Cytochrome P450 (CYP) isoform 3A4 activity, a key enzyme in phase I metabolism. This study examines the remodeling of central and xenobiotic metabolism during the glycine-supplemented differentiation of induced PSCs (iPSCs) into hepatocyte-like cells (HLCs) and HepG2 into a metabolically active form (mHepG2). In these cell systems, glycine promoted oxidative metabolism and mitochondrial function, collagen, glycogen, bile acid anabolism, one-carbon metabolism, and heme biosynthesis, typical of a hepatocyte phenotype. While the metabolic effects of glycine were divergent in mHepG2 and HLCs, in both cases, heme synthesis was boosted by glycine incorporation, a vital feature in supporting xenobiotic metabolism through the heme-containing enzymes CYPs. From this study, a link between glycine supplementation, carbohydrate metabolism, and enhancement of the xenobiotic machinery is established through metabolic plasticity in the maturing hepatocyte.

## Highlights

• High glycine drives the maturation of iPSC into hepatocyte-like cells (HLCs)
• High glycine drives the maturation of HepG2 into metabolically active HepG2 (mHepG2)
• Glycine supplementation improves mitochondrial function in HLCs and mHepG2
• HLCs and mHepG2 acquire CYP activity through glycine-induced heme biosynthesis

## Graphical abstract

**Figure:**
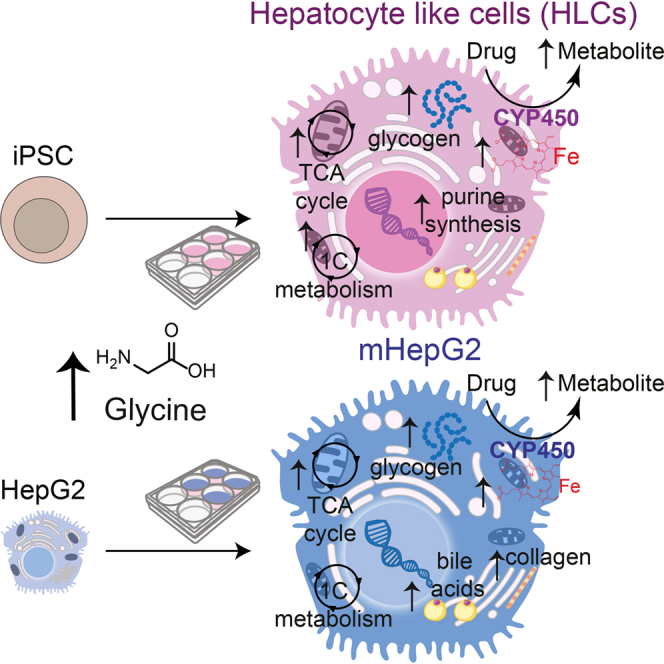

## Introduction

Cellular metabolism is an essential driver in pluripotent stem cells (PSCs) differentiation, pluripotency, and self-renewal maintenance.^1^^,^^2^^,^^3^ Well-defined metabolic changes occur during cellular differentiation from PSCs to differentiated somatic cells. The PSCs' metabolome is characterized by high glycolytic rates, which diminish during stem cell differentiation, alongside an increase in oxidative phosphorylation.^1^ Accordingly, adenosine triphosphate (ATP) production in PSCs is highly dependent on glycolysis, given their immature mitochondria.^2^^,^^3^ Therefore, modulators regulating shifts between glycolysis and oxidative phosphorylation can drive and alter PSC differentiation.^3^

Many studies correlate PSC pluripotency to the glycolytic pathway, but amino acids also take part in stem cell development.^2^^,^^4^^,^^5^ Glutamine (GLN) plays an essential role in PSC survival, generating ATP in the tricarboxylic acid (TCA) cycle, as embryonic stem cells cannot efficiently metabolize pyruvate (PYR) to α-ketoglutarate.^2^ In contrast, GLN metabolism in differentiated cells is not critical due to their capacity to oxidize PYR and lactate (LAC) to produce energy.^2^ Interestingly, this and other metabolic traits are shared between PSCs and cancer cells.^6^ GLN is also the preferred cancer substrate to fuel the TCA cycle.^7^ In addition, cancer cells are characterized by high glycolytic rates followed by LAC production, despite the presence of oxygen - the so-called aerobic glycolysis or Warburg effect - and poor mitochondrial oxidation.^8^ Last, the rapid cell growth observed in both PSCs and cancer cells is supported by high levels of nucleotide production, specifically purine biosynthesis.^9^

Glycine (GLY) is the smallest of amino acids and acts as a precursor in a variety of metabolic pathways, beyond its integral role in protein biosynthesis. It is involved in nucleic acid, creatine, collagen, and porphyrin biosynthesis, as well as being used in conjugation reactions in the antioxidant system.^10^^,^^11^ GLY is easily obtained from the diet, but it is a non-essential amino acid, as it can be synthesized from serine. GLY and serine homeostasis is primarily dealt with in the liver, balancing production and consumption through the enzyme serine hydroxymethyltransferase (SHMT).^12^ Mitochondrial SHMT (SHMT2) is a major GLY-consuming enzyme in the liver, functionally more relevant than the cytosolic SHMT1.^12^ SHMT2 metabolizes GLY and one-carbon (1C) units to produce serine.^12^ Alternatively, GLY is metabolized through the GLY cleavage system (GCS), a multienzyme complex that yields 1C units through its conversion to 5,10-methylene-tetrahydrofolate (5,10-methylene-THF) and CO_2_. 5,10-Methylene-THF can then enter the folate cycle, serving as a central hub for the interconversion of 1C forms. 1C units are key drivers for purine, thymidine, and methionine biosynthesis in the cytosol and are essential for cell growth and development.^9^^,^^12^^,^^13^^,^^14^^,^^15^ However, despite the extensive research on GLY metabolism and its role in cancer cell proliferation,^16^ the mechanism behind GLY in PSC development and hepatic maturation remains elusive.

Cell culture conditions and nutritional environment are pivotal for optimal PSC differentiation.^17^^,^^18^ Boon and colleagues demonstrated that mature hepatocyte-like cells (HLCs) from PSCs can be generated by following a >40-day protocol using media enriched in amino acids, with a high concentration of GLY, exceeding typical nutritional requirements.^4^ HLCs cultured under these conditions were reported to have a metabolome analogous to primary human hepatocytes (PHHs), and increased expression of many hepatic-specific markers and transcriptional pathways.^4^^,^^19^ Furthermore, a comprehensive analysis of the xenobiotic machinery of HLCs was conducted at the transcript, protein, and functional levels for 9 cytochrome P450s, CYP, isoforms. This analysis was benchmarked to PHHs’ performance, concluding the suitability of the HLC model for undergoing drug metabolism studies.^18^ Surprisingly, the hepatocarcinoma cell line, HepG2, cultured under the same nutritional conditions, also acquired enhanced hepatic-like functions. For instance, the activity of various CYP isoenzymes, including the isoform 3A4, CYP3A4, a key enzyme involved in drug metabolism,^20^ increased in the “metabolically matured” HepG2 (mHepG2),^4^^,^^18^ matched with a reduction in glucose (GLU) dependency.^4^

In this study, we combined liquid chromatography mass spectrometry (LC-MS) metabolomics with a cellular respiration assay to characterize the central carbon metabolism (CCM) and mitochondrial function of HLCs, derived from induced PSCs (iPSCs), and mHepG2, along their differentiation. HLCs and mHepG2 were obtained following the high GLY culture conditions described by Boon et al.^4^ GLY supplementation stimulated the activity of various metabolic pathways associated with hepatic-like functions in HLCs and mHepG2. Using a combination of assays, including stable isotope-resolved metabolomics (SIRM), we demonstrated the incorporation of ^13^C_2_-GLY (NH_2_^13^CH_2_COOH) supplementation into porphyrin precursors of HLCs’ heme biosynthesis, driving the drug metabolic capacity obtained in this cell system.

## Results

### Amino acid supplementation - in particular glycine - improves mitochondrial function in mHepG2 and iPSC-derived HLCs

**Figure 1:**
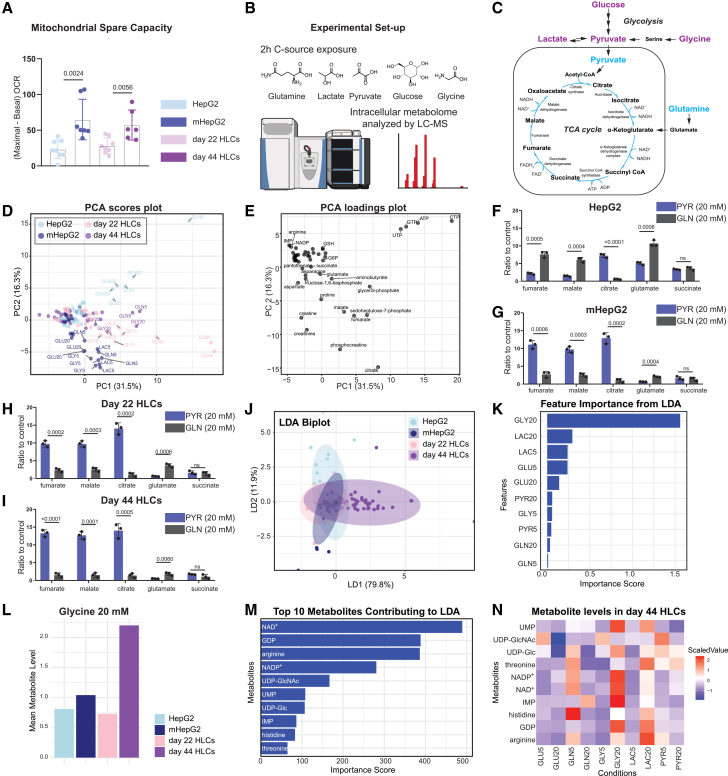
High concentration of amino acids, with 2% (m/v) glycine, increases mitochondrial spare capacity and oxidative metabolism in human liver cell models (A) Mitochondrial spare capacity of the tested liver cell models: HepG2 (light blue), mHepG2 (dark blue), day 22 HLCs (pink), and day 44 HLCs (purple) (N = 6–8, mean ± SD; *t* test *p* value < 0.05 indicated). (B) Scheme of the experimental setup for exposure of carbon sources (2h) in *in vitro* hepatic cell systems for LC-MS semi-targeted metabolomics analysis. (C) Glycolysis and TCA cycle pathways, with highlighted (in color) carbon sources: glutamine (GLN), lactate (LAC), pyruvate (PYR), glucose (GLU), and glycine (GLY). (D) Principal component analysis, PCA (scores plot) and (E) PCA (loadings plot, with selected metabolite names) of cell models’ metabolome (measured by semi-targeted LC-MS) exposed to 5 and 20 mM GLN, LAC, PYR, GLU, and GLY(indicated as GLN5, GLN20, LAC5, LAC20, PYR5, PYR20, GLU5, GLU20, GLY5, and GLY20, respectively), *N* = 3 (metabolite intensities normalized to control, without carbon source). (F–I) Relative abundance of TCA cycle intermediates and glutamate in HepG2 (F), mHepG2 (G), day 22 HLCs (H), and day 44 HLCs (I) incubated with 20 mM PYR (blue) or GLN (gray), (*N* = 3, mean ± SD; metabolite intensities normalized to control; *t* test *p* value < 0.05 indicated; ns, not significant). (J) Linear discriminant analysis (LDA) biplot of cell models’ metabolome measured by semi-targeted LC-MS (HepG2, mHepG2, day 22 HLCs, and day 44 HLCs) incubated with 5 or 20 mM GLN, LAC, PYR, GLU, and GLY, *N* = 3, metabolite intensities normalized to control; ellipses represent 95% statistical confidence. (K) Descending order of the sum of importance scores from the LDA for each condition (*N* = 3). (L) Group average intensities of all measured metabolites across all cell models incubated with 20 mM glycine (GLY20). Metabolite LC-MS intensities were normalized to levels in the control condition (without carbon source). (M) Top 10 metabolites with the highest importance scores from the LDA, considering all conditions and cell models (*N* = 3). (N) Heatmap scaled per row of top 10 metabolites with the highest importance scores in day 44 HLCs incubated with 5 and 20 mM GLN, LAC, PYR, GLU, and GLY, *N* = 3. See metabolite names in Table S1. N: Separate wells of the same differentiation/cell batch independently processed. Extended information in Figures S1, S2, and S3.

Oxidative metabolism is a marker of healthy and differentiated cells. Cancerous cell lines and PSCs are characterized by their high glycolytic rates compared to non-cancerous and somatic cells.^1^^,^^8^ In the context of PSCs differentiation into liver cells, previous research demonstrated that culture conditions with high concentrations of amino acids and 2% (m/v) GLY, far beyond the nutritional requirements, were needed to achieve mature HLCs from PSCs. Surprisingly, similar nutritional conditions “matured” the cancer cell line HepG2 into a more metabolically active cell model, mHepG2, with analogous outcomes.^4^ To better understand this gain-of-function, we sought to characterize the CCM and mitochondrial function of mHepG2 and iPSCs-derived HLCs along their differentiation. For this, HLCs were tested at two stages of differentiation (day 22 and day 44). It was reported previously^4^ that HLCs at day 20 already exhibited a decrease in GLU dependency for energy production, and drug metabolism was further increased at day 40. Immunofluorescence of liver-specific markers was performed, confirming the differentiation of HLCs (Figure S1). The mitochondrial spare capacity (difference between maximal oxygen consumption rate and basal oxygen consumption rate) on day 44 HLCs was significantly increased compared to day 22 HLCs, Figure 1A (and extended Figure S2A). This is an indication of HLC’s further maturation of mitochondrial oxidative metabolism along differentiation, not previously demonstrated.^4^ Likewise, mHepG2 were also compared to HepG2 (after 22 days of maturation) for their mitochondrial function. Again, exposure to high concentrations of amino acids and 2% (m/v) GLY for 22 days increased oxidative capacity in mHepG2 compared to HepG2 cells, Figure 1A (and extended Figure S2A). Confocal microscopy pictures of these 4 cell systems revealed that day 44 HLCs were morphologically closer to PHHs, with some cells being polynuclear, a characteristic of a hepatic phenotype—reproducing previous outcomes.^18^ Furthermore, mHepG2 formed a monolayer, opposite to HepG2, typically occuring in clusters, Figure S2B.

Since mitochondrial function was boosted in day 44 HLCs and mHepG2, we next challenged these cell systems for their carbon source preference. Current medium was depleted, and a single carbon source was provided: GLN, LAC, PYR, GLU, or GLY (at 5 and 20 mM) over 2 h, to evaluate their effect on CCM, Figure 1B. These 5 carbon sources were selected for their contribution within CCM through different metabolic reactions (Figure 1C). As control conditions, all cell models were incubated in Hank’s balanced salt solution (HBSS) without any carbon source. Semi-targeted LC-MS metabolomics was performed with a panel of 64 CCM metabolites^21^ (Table S1). The principal component analysis (PCA) of the normalized (to control conditions) LC-MS data highlighted that each cell system adapted differently to the carbon source, forming cell system-specific clusters, Figure 1D. HepG2 and day 22 HLCs incubated with GLN 5 mM were the conditions introducing the highest variability among the samples, followed by day 44 HLCs incubated with GLN 5 mM and GLY 20 mM. To identify the affected metabolites behind these changes, the corresponding PCA loadings plot, Figure 1E, showed that the nucleotides uridine triphosphate (UTP), guanosine triphosphate (GTP), ATP, and cytidine triphosphate (CTP), as well as the TCA cycle intermediate citrate, carried most of the variance.

HepG2 is a cancerous cell line, and as such, exhibits a high preference for GLN over PYR. Since GLN is crucial in fueling the TCA cycle, it supports the uncontrolled cellular growth by modulating bioenergetics and redox homeostasis.^7^^,^^22^ This is reflected in our results, since HepG2 incorporated GLN into the TCA cycle, leading to increased levels of most TCA cycle intermediates, except for citrate, compared to the PYR condition, Figure 1F. High citrate abundance in HepG2 under PYR incubations may be explained through the mitochondrial citrate-malate shuttle. Translocating citrate to the cytosol fuels fatty acid synthesis, among other cellular processes.^23^ On the contrary, mHepG2 showed the opposite tendency, raising levels of fumarate, malate, and citrate under PYR conditions, compared to GLN, suggesting a more oxidative behavior (Figure 1G).

For both day 22 (Figure 1H) and day 44 (Figure 1I) HLCs, PYR was the preferred carbon source to replenish the TCA cycle, in comparison to GLN. GLN is an essential medium component to cover PSCs' energy requirements, as these cells cannot efficiently oxidize PYR and LAC.^2^ Thus, the differentiation protocol successfully modulated HLCs, already at day 22, toward an oxidative signature, a metabolic feature of differentiated cells.^2^

To further explore the metabolic flexibility of the cell systems, a supervised multivariate statistics model, linear discriminant analysis (LDA), was applied to the normalized dataset to identify the carbon source that maximized the separation between cell models. From the LDA biplot, day 44 HLCs held the most distinct metabolic response when incubated with different carbon sources, compared to HepG2, mHepG2, and day 22 HLCs (Figure 1J). Furthermore, a clear separation between day 22 HLCs and day 44 HLCs was identified. To assess the relative significance of each carbon source in differentiating the cell models, feature importance was calculated based on the squared coefficients of the linear discriminants. By organizing these importance scores in descending order, 20 mM GLY (GLY20), not 5 mM GLY (GLY5), was identified as the most influential carbon source for cell model separation (Figure 1K). The normalized group averages for all measured metabolite intensities revealed that the 20 mM GLY (GLY20) condition had the most cell-type specific response for day 44 HLCs, resulting in a higher metabolite intensity mean, compared to the other hepatic models, Figure 1L (data on other conditions in Figure S3). From the metabolites driving the separation between cell models, predominantly redox cofactors and nucleotides displayed the highest importance scores, Figure 1M. These metabolites increased on day 44 HLCs incubated with 20 mM GLY (GLY20), compared to the other carbon source conditions, Figure 1N. GLY highly contributes to mature HLCs' energy metabolism with a strong activation of biosynthetic pathways.

### Glycine remodels the metabolome of mHepG2 and HLCs

**Figure 2:**
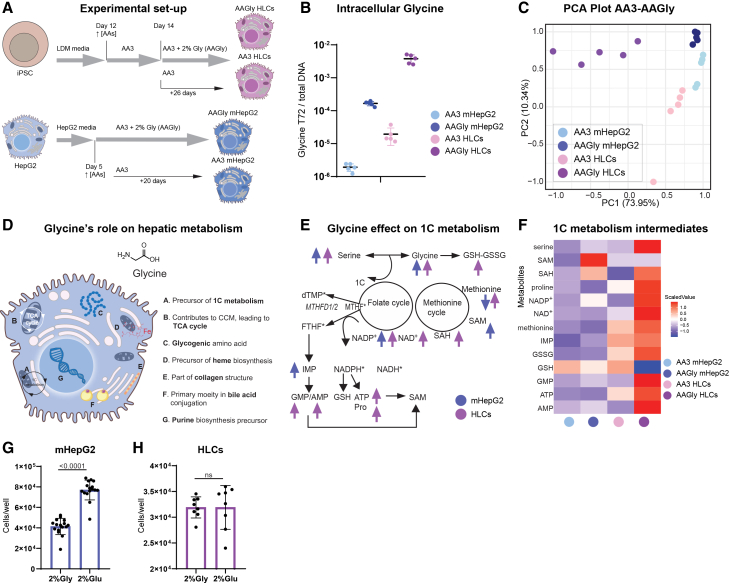
High glycine concentration remodels the metabolome of mHepG2 and HLCs, with the accumulation of 1C metabolism intermediates solely in HLCs (A) Experimental setup of HLCs and mHepG2 differentiation (AA, amino acids; Gly, glycine). (B) Intracellular glycine content in mHepG2 (blue) and HLCs (pink), cultured in AA3 (light) and AAGly (dark), after 72 h (glycine LC-MS intensity normalized by total DNA amount (μg), mean ± SD, *N* = 5). (C) Scaled and centered PCA (scores plot) of mHepG2 and HLCs differentiated with and without 2% (m/v) glycine (*N* = 5, LC-MS metabolite intensities normalized by total DNA (μg) per sample). (D) Glycine’s metabolic role in the hepatocyte. (E) Comparison of levels of one-carbon metabolism intermediates in the two tested liver models under AA3 and AAGly conditions (↑ represents a significant metabolite increase in the AAGly condition compared to AA3, in HLCs (purple) and mHepG2 (blue); *N* = 5; ∗ metabolite not measured). (F) Heatmap of 1C metabolites obtained by LC-MS, for AA3 and AAGly conditions in mHepG2 and HLCs (*N* = 5; data normalized to total DNA (μg), and scaled per row using min-max function). (G and H) Number of cells per well after 20 days (mHepG2) (G) and 40 days (HLCs) (H) of culture in AA3 with 2% (m/v) glycine (Gly) or 2% (m/v) glucose (Glu), *N* = 16, 8; mean ± SD. All statistical differences presented in the figure were assessed using an unpaired *t* test, indicated for *p* value < 0.05; ns: not significant. N: Separate wells of the same differentiation batch independently processed. Extended information in Figure S4.

To assess the role of GLY on hepatic maturation, day 44 HLCs and mHepG2 were differentiated under two conditions: media enriched in amino acids, with 2% (m/v) GLY (AAGly condition, 268 mM final GLY concentration) or media enriched in amino acids, without 2% (m/v) GLY (AA3-condition, 1.5 mM final GLY concentration), Figure 2A.

After differentiation under these two conditions (AA3 and AAGly), and following a 72-h feeding period, intracellular GLY levels were measured by LC-MS, showing an expected higher abundance in AAGly compared to AA3 conditions, and no GLY depletion in any of the groups (Figure 2B). This suggested a role for GLY beyond a simple nutritional requirement. The metabolome of both cell models (HLCs and mHepG2) under the two conditions (AA3 and AAGly) was analyzed using semi-targeted LC-MS metabolomics (see Table S1). From the PCA plots of the metabolome data, a profound remodeling of the metabolome under AAGly was identified in mHepG2 and, to an even larger extent, in HLCs compared to AA3 (Figure 2C). This led us to hypothesize that GLY supplementation was key in eliciting the metabolic shift required for differentiating the cell systems into a more hepatic-like status.

GLY is involved in several hepatic metabolic pathways, Figure 2D: TCA cycle, gluconeogenesis, heme biosynthesis, collagen biosynthesis, bile acid anabolism, and purine biosynthesis.^9^^,^^12^^,^^24^^,^^25^^,^^26^^,^^27^ GLY is connected to 1C metabolism through serine conversion by the enzyme SHMT and through the GCS.^9^^,^^12^ The mitochondrial SHMT (SHMT2) reaction is reversible, either from GLY to serine, consuming 1C units, or from serine to GLY, producing 1C units.^12^ Due to GLY’s role in 1C metabolism, we further investigated differences in pathway intermediate levels between the two cultured conditions (AA3 and AAGly). On AAGly HLCs, high GLY concentration increased most of the 1C intermediates, Figures 2E and S4. Surprisingly, high GLY concentration did not have the same effect in mHepG2, where only serine, nicotinamide adenine dinucleotide phosphate (NADP^+^), inosine monophosphate (IMP), and *S*-adenosyl methionine (SAM) showed a significant increase in the AAGly condition compared to AA3, Figure 2E. Among all conditions, 1C intermediates were highly abundant in HLCs incubated under high concentrations of GLY, except for SAM and reduced glutathione, GSH, Figures 2F and S4. Notably, and supporting previous results (Figure 1N), the redox cofactors NADP^+^ and NAD^+^ were boosted in HLCs under high GLY concentration, suggesting an upregulation of oxidative metabolism.

Increased 1C metabolism rates are associated with cancer cells’ rapid growth.^9^ To assess the effect of high GLY concentration on cell proliferation, mHepG2 and HLCs were incubated with AA3 media supplemented with either 2% (m/v) GLY or 2% (m/v) GLU. Interestingly, GLY supplementation significantly reduced mHepG2’s cell number compared to GLU supplementation (Figure 2G). In HLCs, the two carbon sources led to an equal number of cells, Figure 2H. These results reinforce previous data, Figure 2E, demonstrating that high concentrations of GLY do not support 1C metabolism in mHepG2, consequently reducing cell number.

### Glycine supplementation supports glycogen storage, collagen biosynthesis, and bile acid anabolism

Due to the significant effect of GLY on hepatic metabolome, we further investigated its effect on cell energy storage (in the form of glycogen),^28^ collagen biosynthesis,^27^ and bile acid anabolism.^26^ All three of these metabolic pathways are essential functions of the liver.

**Figure 3:**
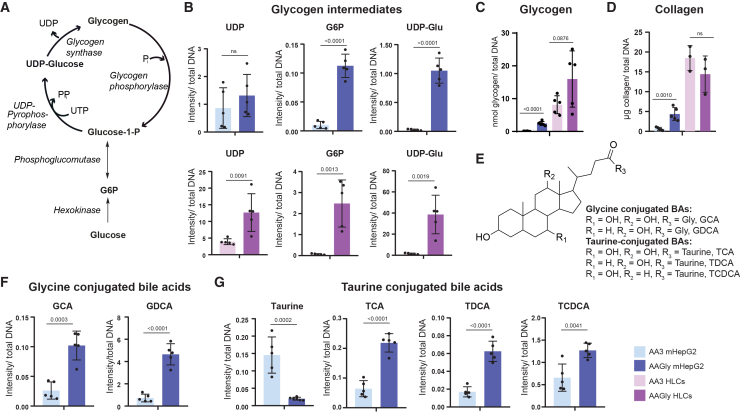
Glycine supplementation increases glycogen, collagen, and bile acid biosynthesis (A) Scheme of glycogen biosynthesis. (B) Glycogen intermediates measured by LC-MS in mHepG2 and HLCs with (AAGly) and without (AA3) 2% (m/v) glycine (metabolite intensity normalized to total DNA in μg; *N* = 5). (C) Glycogen amount (nmol) normalized to total DNA in μg for mHepG2 and HLCs under AA3 and AAGly conditions (*N* = 6,5). (D) Effect of high concentration of glycine on collagen content (μg collagen/μg DNA, *N* = 5,3). (E) Main structure of bile acids (BAs), glycine or taurine substituted. (F and G) High glycine concentration increases both glycine (F) and taurine-conjugated (G) BAs (putative metabolite assignment by LC-MS; metabolite intensity normalized by total DNA in μg; *N* = 5). In panels B–D, F and G, AA3 mHepG2 (light blue), AAGly mHepG2 (dark blue), AA3 HLCs (pink), and AAGly HLCs (purple); replicates represented as individual dots, mean ± SD. Statistical differences assessed by unpaired *t* test, considering significance for depicted *p* value < 0.05; ns, not significant. Metabolite names in Tables S1 and S2. N: Separate wells of the same differentiation batch independently processed.

GLY is a glucogenic amino acid, and its conversion to PYR enhances glucose-6-phosphate (G6P) and uridine diphosphate glucose (UDP-Glu) levels, ultimately promoting glycogenesis via gluconeogenesis,^28^ Figure 3A. HLCs and mHepG2 cultured with AAGly significantly increased glycogen synthesis intermediates, Figure 3B, resulting in increased glycogen levels (or an increased trend in HLCs), Figure 3C, compared to AA3 conditions.

Next, we assessed whether GLY would promote collagen biosynthesis. Collagen contains triple-helical domains, which repeatedly hold a core sequence of G-X-Y primary structure. G resembles GLY, and X and Y can represent any amino acid, often covered by proline and hydroxyproline, respectively. All collagens contain at least one triple-helical domain.^29^ Collagen is an essential component of the extracellular matrix (ECM) of hepatocytes, supporting the differentiation and polarization of cells.^27^ High GLY concentration significantly increased the amount of soluble collagen in mHepG2, but it did not significantly affect collagen content in HLCs, Figure 3D. This result is corroborated by the lower proline amount detected in AAGly mHepG2, compared to AA3 mHepG2 (Figure S4).

Last, since GLY is one of the amino acids involved in bile acid anabolism,^26^ relative amounts of the GLY and taurine conjugated bile acids (Figure 3E), putatively identified as glycocholic acid (GCA), glycodeoxycholic acid (GDCA), taurocholic acid (TCA), taurodeoxycholic acid (TDCA), and taurochenodeoxycholic acid (TCDCA), were analyzed by LC-MS in the two *in vitro* systems, but were only detected in mHepG2 (metabolite details in Table S2). Interestingly, higher amounts of bile acids, both GLY and taurine conjugated, were found under culture conditions with 2% (m/v) GLY (AAGly), Figures 3F and 3G. However, taurine levels were decreased (Figure 3G), possibly indicating a GLY-induced stimulation of taurine conjugation to bile acids through bile acid-CoA: amino acid *N*-acyltransferase (BAAT).

### Glycine promotes the expression of heme biosynthetic enzymes and CYP activity

It was previously reported that 2% (m/v) GLY culturing conditions increased the maturation of HLCs and mHepG2 with concomitant expression and activity of several cytochrome P450 isoforms, with particular interest in CYP3A4 due to its relevance in drug metabolism.^4^^,^^18^ The involvement of mTOR was previously proposed as the biochemical mechanism behind it.^4^ However, this argument did not completely explain why GLY significantly improved CYP3A4 activity more than other amino acids previously tested (alanine, serine, or leucine).^4^

**Figure 4:**
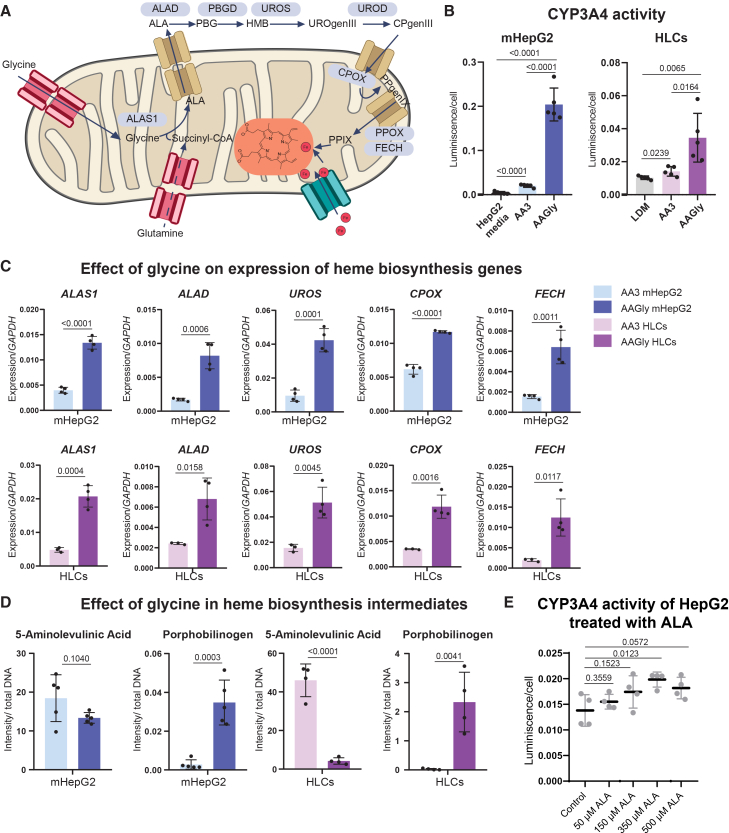
Glycine increases the expression of heme biosynthesis genes, heme intermediates and CYP3A4 activity in mHepG2 and HLCs (A) Simplified scheme of the heme biosynthesis pathway inside and outside the mitochondrial matrix (adapted from Yien & Perfetto, 2022). (B) CYP3A4 activity measured with the Glo-Assay (luminescence, normalized by cell count) of mHepG2 and HLCs under the condition of basal culture media (in gray: HepG2 culture media for mHepG2 and LDM for HLCs), AA3 (light blue-AA3 mHepG2, pink-AA3 HLCs) and AAGly (dark blue-AAGly mHepG2, purple-AAGly HLCs), *N* = 5. (C) Increased gene expression of heme biosynthesis enzymes (*ALAS1*, *ALAD*, *UROS*, *CROX*, and *FECH*, relative to the housekeeping gene *GAPDH*) by the presence of high glycine concentration in the culture media (*N* = 3,4; see Table S3). (D) Heme biosynthesis intermediates measured by LC-MS (Table S2). Metabolite intensity normalized by total DNA in μg (*N* = 5,4). (E) HepG2 CYP3A4 activity, measured with the Glo assay, after 72 h exposure to 0 (control), 50, 150, 350, and 500 μM 5-aminolevulinic acid (ALA), *N* = 4. For panels B–E, statistical differences assessed by unpaired *t* test, and considered significant for depicted *p* value < 0.05, replicates represented as individual dots, mean ± SD. N: Separate wells of the same differentiation batch independently processed. Enzyme and metabolite names: ALAS1, 5-aminolevulinic acid synthase 1; ALAD, 5-aminolevulinic acid dehydratase; PBGD, porphobilinogen deaminase; UROS, uroporphyrinogen III synthase; UROD, uroporphyrinogen III decarboxylase; CROX, coproporphyrinogen oxidase; FECH, ferrochelatase; PPOX, protoporphyrinogen oxidase; ALA, 5-aminolevulinic acid; PBG, porphobilinogen; HMB, hydroxymethylbilane; UROgenIII, uroporphyrinogen III; CPgenIII, coproporphyrinogen III; PPgenIX, protoporphyrin.

Here, we investigated whether excess GLY supplementation further increased heme biosynthesis. CYP3A4 is a heme-containing protein.^30^ Therefore, we hypothesized that high levels of GLY and increased CYP3A4 activity were connected through the stimulation of heme anabolism during differentiation. GLY, inside the hepatic mitochondria, reacts with succinyl-CoA to form 5-aminolevulinic acid (ALA) by the enzyme ALA synthase 1, ALAS1. ALA is then released to the cytosol and converted to porphobilinogen (PBG) by ALA dehydratase (ALAD), Figure 4A.^25^ PBG, through consecutive reactions taking place in the cytosol, is finally metabolized to protoporphyrin IX, which, back in the mitochondrial matrix, is conjugated with ferrous ions to form heme complexes.^25^ About 15% of the body’s heme daily production takes place in hepatocytes for the formation of heme-containing proteins.^31^

In this study, we reproduced previous findings^4^^,^^18^: high concentration of GLY in the culture media did increase CYP3A4 activity in mHepG2 and HLCs, Figure 4B. The expression of several enzymes of the heme biosynthesis pathway: *ALAS1*, *ALAD*, uroporphyrinogen III synthase (*UROS*), coproporphyrinogen oxidase (*CROX*), and ferrochelatase (*FECH*) was semi-quantified by qPCR, Figure 4C. GLY clearly increased the expression of all tested mRNAs involved in the heme biosynthesis pathway for both mHepG2 and HLCs. Moreover, PBG levels as determined by LC-MS were significantly higher in AAGly for both HLCs and mHepG2 (Figure 4D). In contrast, ALA levels showed a slight reduction (mHepG2) or a substantial reduction (HLCs) in AAGly versus AA3. Increased expression of all biosynthesis genes and higher levels of PBG indicate an enhanced heme anabolism due to GLY supplementation. Lower ALA levels found in the AAGly conditions may be attributed to the known negative feedback regulation of ALAS1^32^ or due to the reaction’s stoichiometry, as two molecules of ALA yield one of PBG, through ALAD.^25^

To further test whether heme biosynthesis is a limiting factor in CYP3A4 assembly and thus, activity, HepG2 cells were treated with increasing concentrations of ALA (Figure 4E). HepG2 cells, known for their low CYP3A4 expression,^4^ when administered with ALA, showed a progressive tendency to increase CYP3A4 activity compared to control conditions (Figure 4E). The highest CYP3A4 activity was achieved at 350 μM ALA. These results further confirm the role of GLY in increasing heme formation, coining an immature heme synthesis as an underlying factor in HepG2’s low CYP3A4 activity.

### ^13^C_2_-glycine supplementation promotes 1C and heme metabolism in HLCs

**Figure 5:**
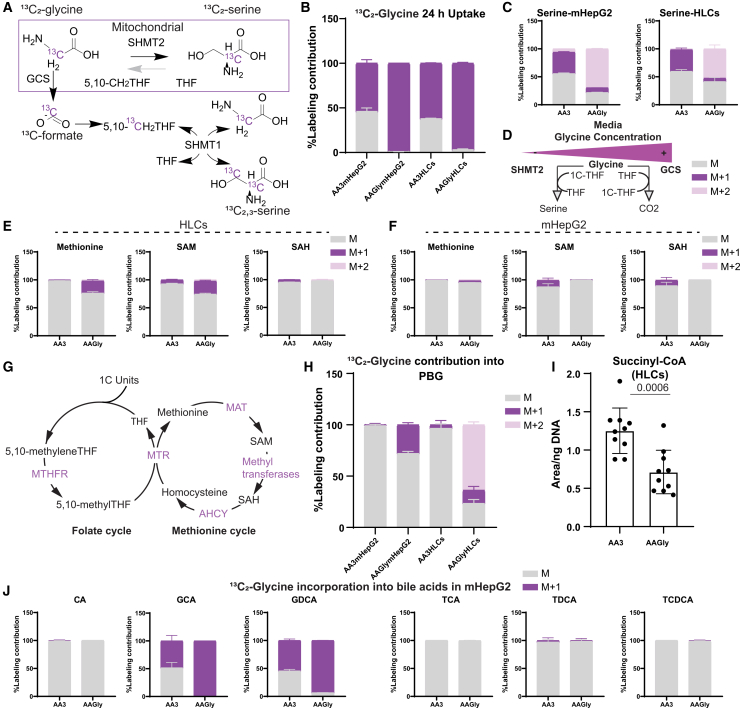
High and low glycine concentrations result in distinct glycine homeostasis in mHepG2 and HLCs LC-MS based stable isotope ratio metabolomics (SIRM) measured the labeling incorporation from ^13^C_2_-glycine into various intracellular metabolites: isotopologues M, unlabeled fraction (gray); M+1, one labeled carbon (purple); and M+2, two labeled carbons (pink) (mean ± SD, *N* = 5–6). (A) Scheme of ^13^C_2_-glycine-metabolism to serine through mitochondrial SHMT2 and GCS. (B) Intracellular ^13^C_2_-glycine incorporation after 24 h exposure in mHepG2 and HLCs under low (AA3) and high (AAGly) concentrations. (C) Labeling contribution of ^13^C_2_-glycine on serine in mHepG2 and HLCs differentiated under AA3 and AAGly conditions. (D) Scheme of glycine homeostasis under low and high concentrations. (E and F) Labeling contribution of ^13^C_2_-glycine on methionine cycle intermediates: methionine, SAM, and SAH, in (E) HLCs and (F) mHepG2 differentiated under AA3 and AAGly conditions. (G) Scheme of folate and methionine cycle. (H) ^13^C_2_-Glycine incorporation into the heme intermediate PBG. (I) Levels of unlabeled succinyl-CoA measured by LC-MS comparing AA3 and AAGly HLCs conditions (mean ± SD, *N* = 10, LC-MS intensity normalized by total DNA, in ng; *t* test *p* value < 0.05 indicated). (J) ^13^C_2_-Glycine incorporation into putatively identified bile acids in mHepG2. N: Separate wells of the same differentiation batch independently processed. Enzyme and metabolite names: 5,10-CH_2_THF, 5,10-methylenetetrahydrofolate; THF, tetrahydrofolate; GCS, glycine cleavage system; SHMT, serine hydroxymethyltransferase; MAT, methionine adenosyltransferase; MTHFR, methylenetetrahydrofolate reductase; MTR, methionine synthase; AHCY, adenosylhomocysteinase (S-adenosylhomocysteine hydrolase).

Inside the cell, GLY is metabolized through two primary routes: the cytosolic or mitochondrial SHMT1/2 (reverse flux), consuming 1C units and producing serine, or alternatively through the GCS, leading to the formation of cytosolic formate.^12^^,^^14^ Formate can then re-enter 1C-metabolism in the cytosol, generating serine from GLY,^14^ Figure 5A. To further explore GLY homeostasis in HLCs and mHepG2, under low and high concentrations, and its implication over the studied metabolic pathways, we performed isotope tracing using ^13^C_2_-GLY (NH_2_^13^CH_2_COOH). Both HLCs and mHepG2, differentiated under AA3 and AAGly conditions, were exposed to isotopically labeled GLY using the same concentration as their respective culture conditions (1.5 and 268 mM) for 24 h, one day after differentiation.

After 24 h, cells (HLCs and mHepG2) cultured under a high concentration of GLY (AAGly) replaced, almost to 100%, intracellular GLY with extracellular ^13^C_2_-GLY, Figure 5B. The low GLY condition (AA3) incorporated merely ∼55% in either cell system. This may indicate either a higher uptake or a higher intracellular GLY utilization and metabolism in the AAGly condition. Given the reversibility of the SHMT1/2 reactions, we then measured label incorporation into intracellular serine. In the low GLY condition, ∼40% of intracellular serine was labeled, M+1 (^13^C_2_-serine) in both cell models, obtained via the SHMT2 reaction, Figure 5C. However, in the high GLY condition, ∼50% (in HLCs) or ∼70% (in mHepG2) of detected serine was M+2 (^13^C_2,3_-serine), formed via the SHMT1 reaction, Figure 5C. In the AAGly condition, ^13^C_2,3_-serine was formed through the 5,10-methylene-THF pool,^14^ suggesting GLY metabolism through GCS. Thus, higher GLY concentration, in both HLCs and mHepG2, enhanced GCS activity (Figure 5D).

To further explore this difference in GLY utilization, under the two culture conditions, label incorporation in 1C intermediates was measured. In HLCs, ∼20% labeled M+1 fractions were detected in methionine and SAM under the AAGly condition, but not under the AA3 condition (Figure 5E). Label incorporation into 1C intermediates in HLCs is likely to be taken via the formed ^13^C-formate generated through GCS ^13^C_2_-GLY metabolism. This may explain the absence of a label in *S*-adenosyl-L-homocysteine (SAH), as the ^13^C-carbon is transferred during the SAM-SAH conversion in methylation reactions. However, in mHepG2, high GLY concentration did not significantly support methionine cycle intermediates, Figures 5F and 5G. This is in line with our previous observation that a high concentration of GLY only led to the accumulation of 1C intermediates in HLCs, and not in mHepG2 (Figure 2). In fact, high GLY concentration decreased methionine levels in mHepG2 (Figure S4).

Given the mild label incorporation in 1C metabolites, labeling patterns of other GLY-related pathways were researched. ^13^C_2_-GLY contributed to the porphyrin precursor PBG in both mHepG2 and HLCs AAGly conditions (Figure 5H). Specifically, PBG M+2 showed the highest contribution in AAGly HLCs (two molecules of ALA M+1 yield one molecule of PBG, resulting in M+2 labeling). Supporting this, levels of unlabeled succinyl-CoA were measured in the HLCs samples, showing lower metabolite abundance in the AAGly condition compared to AA3, Figure 5I. Succinyl-CoA and GLY form ALA by ALAS1; thus, a higher concentration of GLY drives this reaction, consuming succinyl-CoA. In contrast, labeling patterns of GLY-conjugated bile acids (GCA and GDCA) showed elevated ^13^C_2_-GLY incorporation in mHepG2 (Figure 5J). As expected, almost no labeling was incorporated into the taurine-conjugated bile acids (TCA, TCDA, and TCDCA).

Through SIRM, the metabolic link between GLY, 1C metabolism, and heme biosynthesis was strengthened, ultimately leading to the development of the xenobiotic machinery in the maturing hepatocyte.

### Glycine-associated metabolic pathways are downregulated in patients with hepatocarcinoma

**Figure 6:**
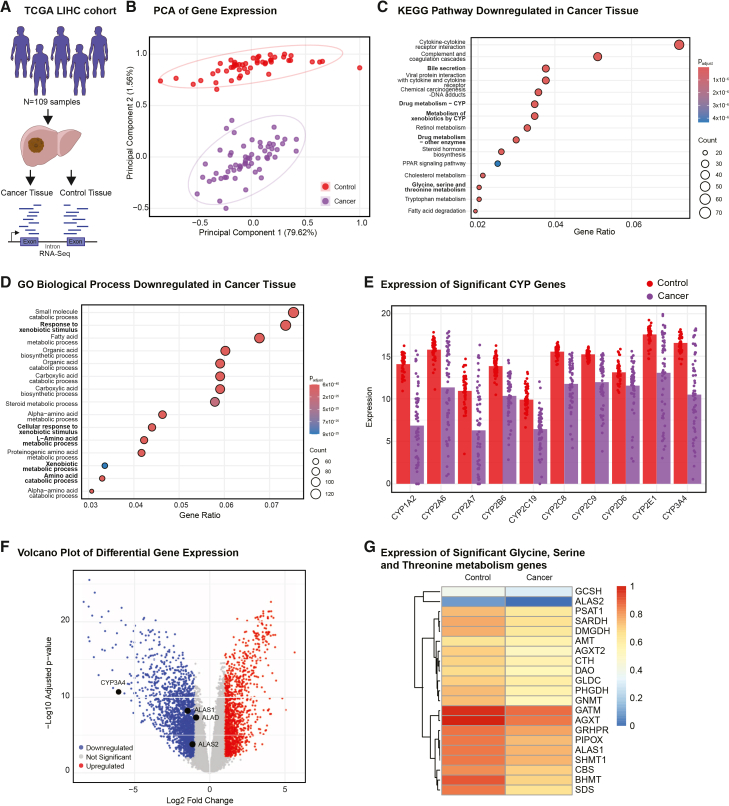
Glycine-stimulated pathways are significantly downregulated in liver hepatocellular carcinoma samples in patients of the LIHC cohort Analysis of the publicly available LIHC RNA-Seq dataset, containing 109 samples. (A) Scheme of the LIHC cohort. (B) Principal component analysis, PCA (scores plot) of control (red) and cancer (purple) tissue samples. Data were centered and scaled using the min-max function. (C) KEGG pathway enrichment analysis of significantly downregulated genes in cancer samples. (D) GO biological process enrichment analysis of significantly downregulated genes in cancer samples. (E) Expression of significant CYP450 genes differentially expressed in control (red) and cancer (purple) samples. (F) Volcano plot of differential gene expression indicating significantly (*p* value < 0.01) downregulated (blue) and upregulated (red) genes. (G) Heatmap expression of significantly differentially expressed genes involved in glycine, serine, and threonine metabolism. Data were normalized using the min-max function. Extended information in Figure S5.

Due to GLY’s implications in health and disease,^13^^,^^16^^,^^24^^,^^33^ and since the liver is the main organ in charge of serine and GLY homeostasis,^12^ we were curious to assess whether this link between GLY and xenobiotic metabolism would hold true in a clinical setting. To this end, we analyzed the public dataset from the TCGA liver hepatocellular carcinoma cohort (LIHC), Figure 6A. We hypothesized that hepatocarcinoma tissue is likely to have impaired xenobiotic metabolism machinery, namely CYP activity, mirroring our findings in HepG2. From the PCA of the LIHC gene expression data, a clear separation between control and cancer tissue from patients was evident (Figure 6B). Interestingly, when performing pathway analysis on the differentially expressed genes, xenobiotic metabolism appeared significantly downregulated in both KEGG^34^ and Gene Ontology (GO) biological process^35^ pathway enrichment analysis, Figures 6C, 6D, and S5. Specifically, within drug metabolism, CYP isoforms were significantly lowered in the cancer tissue of patients (Figure 6E). The first enzymes involved in heme biosynthesis (ALAS1, the erythroid-specific isoform ALAS2, and ALAD) were also reduced in cancer tissue samples, Figure 6F. Bile secretion, GLY, serine, and threonine metabolism, all GLY-related pathways, were among the top 15 KEGG downregulated pathways, Figure 6C. Distinctively, SHMT1, GLY decarboxylase (GLDC), and GLY *N*-methyltransferase (GNMT), involved in GLY metabolism, appeared significantly decreased, Figure 6G.

These clinical results demonstrate that xenobiotic metabolism, amino acid metabolism, and bile acid biosynthesis were downregulated in patients with hepatocellular carcinoma. These findings mirror our observations in HepG2, as similar pathways are stimulated upon GLY supplementation. This not only confirms our mechanistic findings in the role of GLY in hepatic metabolism but also suggests a potential translational arm into clinical settings.

## Discussion

Metabolism is a hallmark of living systems, with the cell metabolome serving as a unique fingerprint of distinct cellular developmental states. Boon et al.^4^ reported that an environment with a high concentration of GLY, beyond the nutritional requirement, is needed to mature stem cells into HLCs, as well as HepG2 into mHepG2. These elevated levels of GLY were able to sustain cells in culture for >50 days and provided them with xenobiotic activity, as tested on a panel of pharmaceutical drugs.^4^^,^^18^ With lower GLU dependency, expression of hepatic markers, and CYP enzymatic activity, these cell models showed promise to perform as functional *in vitro* human hepatocytes. The mechanism behind GLY supplementation was attributed to the mTORC1 regulation of *CYP* expression.^4^ mTOR is a known master regulator of metabolism, responding to nutrients, such as amino acids.^4^ Moreover, mTORC1 influenced mitochondrial activity and oxidative phosphorylation^4^^,^^36^ in PSCs. However, activation through mTOR alone could not fully explain why GLY, more than other tested amino acids, led to hepatic maturation. Leucine and arginine remain the prime amino acid stimulators of this pathway.^37^^,^^38^

In our study, we adapted the reported protocol^4^ to produce metabolically performing *in vitro* human hepatic cells: HLCs and mHepG2. Our findings support that culture conditions with a high concentration of amino acids, specifically GLY (well above physiological conditions 0.17–0.33 mM^39^), induced hepatic maturation in HLCs and mHepG2.^4^^,^^19^ First, bioenergetics and mitochondrial function were studied. Both cell systems became more oxidative and less glycolytic along differentiation. Cell CCM maturation was time-dependent, as HLCs presented higher mitochondrial spare capacity at later stages of differentiation compared to earlier stages. Further, the CCM of HLCs and mHepG2 showed that in later stages of the differentiation, a high concentration of GLY led to a significant remodeling of the hepatic metabolome. Matured HLCs made use of GLY not only to cover energy demands but also to promote developing biosynthetic pathways.

The metabolic changes observed in cells supplemented with different concentrations of GLY (high and low) prompted us to assess cellular GLY homeostasis. For HLCs and mHepG2 under low GLY concentration (AA3 condition), the reverse flux of SHMT (GLY conversion to serine) was the preferred route to deal with GLY content, decreasing 1C availability. Conversely, under high concentrations of GLY, GCS was the predominant route of GLY metabolism.^40^ Thus, high GLY concentration reduced the consumption of 1C units through the reverse SHMT2 flux, increasing the cytosolic 1C pool through GCS stimulation. However, semi-targeted metabolomics revealed that 1C metabolism intermediates accumulated in HLCs, but not in mHepG2 under these high GLY conditions. These results align with previous research, demonstrating that GLY, in cancerous cells, does not support 1C metabolism, thereby inhibiting their proliferation.^33^ According to our data, 1C units generated through GCS under high GLY conditions, in mHepG2, are further utilized for serine production via SHTM1 (reverse) flux, rather than entering folate-methionine cycles, as observed in HLCs. Changes in the 1C pool may be a contributor to mHepG2’s reduced proliferation and overall observed metabolic shift.^7^^,^^41^ Distinct patterns in GLY utilization by HLCs and mHepG2 may be further attributed to HepG2’s low content and poorly developed mitochondria.^42^ On the other hand, HLCs under high GLY conditions increased purine biosynthesis, essential for regulating gene expression, energy production, and RNA synthesis, in line with previous reports on stem cell development.^2^^,^^9^ A boost in methyl donors through 1C metabolism (SAM-SAH conversion) drives epigenetic programs, which were reported to be essential in neural^43^ and kidney^44^ cell fate differentiation.

In this study, we demonstrated that GLY plays a crucial role in supporting energy homeostasis in hepatic cells *in vitro*, enhancing glycogen levels by stimulating gluconeogenesis in both mHepG2 and HLCs. This is clinically relevant because previous studies have shown that the levels of GNMT, the liver’s most prevalent methyltransferase, are downregulated in hepatocellular carcinoma cells, resulting in decreased gluconeogenesis, as evidenced by experiments on GNMT knockout mice.^45^ Moreover, high GLY concentration increased mHepG2 collagen content, highlighting its contribution as a hepatic structural scaffold, since hepatoma cells present an altered ECM composition.^46^ For HLCs, GLY did not affect collagen levels, as these cells were cultured on Geltrex-coated plates that were already supplemented with different polymers.

The expression of all tested enzymes involved in heme biosynthesis was significantly increased in mHepG2 and HLCs under a nutritional regimen with high GLY concentration. Using SIRM, ^13^C_2_-GLY direct incorporation was found in PBG, the precursor of porphyrins. Thus, a higher expression in heme biosynthesis enzymes and precursor availability increased heme complexes, resulting in higher translation and functionality of CYP450, heme-monooxygenase proteins.^30^ Analogous results were reported by Islam et al.^30^ Hemin, ferric chloride heme, increased CYP3A4 activity of HepG2 cells pre-stimulated with rifampicin,^30^ indicating that heme complexes are limiting for CYP3A4 function. Furthermore, similar strategies were used by Kim et al., who used iron ions to mature PSC-derived hepatic organoids, resulting in higher CYP3A4 activity.^47^ We and others have previously reported that GLY supplementation, under the same culture conditions used in this study, led to activity of not only CYP3A4/5, but also other CYP isoforms.^4^^,^^18^ CYP1A2, CYP2A6, CYP2B6, CYP2C8, CYP2C9, CYP2D6, and CYP2E1 expression and activity were also obtained in mHepG2 and/or day 44 HLCs (higher than in HepG2 and day 22 HLCs, respectively).^4^^,^^18^ Heme synthesis through GLY supplementation stimulates the activity of heme containing enzymes, and by extension, phase I drug metabolism.

Metabolic modulation has been utilized in promoting stem cell differentiation.^48^^,^^49^ The induction of the so-called “stem cell differentiation triggers,” such as mitochondrial biogenesis, shift to oxidative phosphorylation, and downregulation of glycolysis, has been reported in other studies to promote cardiomyocyte and neuronal differentiation.^48^ Nevertheless, the use of high concentrations of GLY in regimens for differentiating other cell types has yet to be investigated. Alternative protocols, excluding high-amino acid supplementation, have been shown to produce HLCs that exhibit similar metabolic switches during their differentiation.^50^ These include heightened oxidative phosphorylation, reduced GLN and GLU dependency, and notably, a significant consumption of amino acids such as methionine, cystine, histidine, lysine, valine, and isoleucine, with increasing rates toward later stages of differentiation.^50^ Amino acid supplementation is then suggested to potentiate hepatic characteristics in analogous protocols. Other PSC donors were used to generate HLCs, applying the same differentiation protocol as the one utilized in our study.^4^^,^^51^ Donor-specific metabolic traits were obtained in the mature hepatocytes with slightly different metabolic activities (e.g., variation in CYP3A4 functional levels).^4^^,^^51^ This repeatability using different donors confirmed the reproducibility of the culture protocol. Remarkably, the culture conditions used in the present study reduced cancerous metabolic features of mHepG2, including reduced proliferation rates and increased mitochondrial spare capacity and oxidative behavior, shifting carbon source preference from GLN to PYR. This feat not only achieved a more hepatocyte-like *in vitro* model but also demonstrates the potential to alter the metabolic characteristics of cancerous cells through nutrient supplementation.

Finally, pathway enrichment analysis of the TCGA LIHC cohort RNA-Seq data revealed that genes involved in xenobiotic metabolism are downregulated in patients with liver hepatocellular carcinoma. Bile acid secretion, another GLY-stimulated pathway, ranked among the top 15 KEGG downregulated pathways, alongside enzymes associated with GLY, serine, and threonine metabolism (GMNT, SHMT1, and the GCS enzyme GLDC, among others). Our study revealed that high GLY culture conditions diminished metabolic carcinogenic traits in HepG2 cells and enhanced pathways known to be downregulated in liver cancer tissue.

Xenobiotic and carbohydrate metabolism, although not often studied together, have been previously linked and associated with health and disease. High reverse flux of SHTM2 in hepatocytes (from GLY to serine) is related to metabolic dysfunction-associated steatosis liver disease (MASLD), as a cause of GLY depletion.^52^ Ghrayeb and colleagues linked low GLY levels to low GSH abundance in MASLD, which induced hypersensitivity to acetaminophen toxicity through increased reactive oxygen species production.^52^ The authors further propose targeting SHMT2 as a therapeutic strategy to improve xenobiotic detoxification *in vivo*.^52^ A concurring mechanism in MASLD may be proposed here, as reduced CYP translation (through decreased heme synthesis) may be a consequence of the GLY deficiency.

In conclusion, this study elucidated the mechanistic and multifaceted role of GLY in the differentiation of iPSCs into hepatocytes, largely driven by metabolic processes. While alternative signaling effects may not be completely discarded, this study coins GLY as a major metabolic driver in hepatocyte maturation. GLY supplementation also showed potential in altering cancerous metabolic characteristics of hepatocarcinoma cells (HepG2), toward improved functionality. This study represents a promise in the use of metabolic modulation for advancing the development of iPSCs strategies into higher translatability of *in vitro* systems of undeniable relevance in future drug development fields (ADME: absorption, distribution, metabolism, and excretion and DMPK: drug metabolism and pharmacokinetics) and clinical applications at large.

### Limitations of the study

Although this study elucidates the role of GLY in *in vitro* hepatic maturation and function, it remains elusive whether GLY will cause the same effect *in vivo*. GLY was shown to remodel the metabolome of the hepatocellular carcinoma cell line (HepG2), enhancing its metabolic function, in particular CYP3A4, through the stimulation of heme biosynthesis. The same association between GLY, heme, and CYP activity was found in a human cohort (LIHC); nevertheless, the causality remains elusive, and dedicated *in vivo* and/or clinical studies would have to assess its validity.

Moreover, this study is limited by its unique metabolic perspective in assessing GLY’s role, as major promotor of metabolic activity. As previously suggested,^4^ GLY’s contribution may be relevant at a signaling level, as much of the hepatic differentiation is driven by other cellular processes, such as transcription factor involvement, beyond metabolism. For that, the intersection between the metabolic and transcriptional layers could further complement the effects of GLY in the maturing hepatocyte.

Last, the semi-quantification of certain intermediates of the heme biosynthesis pathway (by LC-MS) was technically challenging, limiting the identification of intermediates to 5-ALA and PBG. Dedicated targeted LC-MS methods for quantifying downstream heme intermediates would further strengthen the data reported here.

## Resource availability

### Lead contact

Requests for further information, resources, and reagents should be directed to and will be fulfilled by the lead contact, Sofia Moco (s.moco@vu.nl).

### Materials availability

This study did not generate new unique reagents.

### Data and code availability

• All metabolomics data generated in this study (Figures 1, 2, 3, and 5) were deposited in the National Metabolomics Data Repository^53^ (NMDR; https://www.metabolomicsworkbench.org), and processed data acquired from qPCR and Seahorse bioanalyzer (Figures 1 and 4) were deposited in Zenodo.^54^
• Figure 1 Cellular respiration data (Seahorse): https://doi.org/10.5281/zenodo.19162793; Figure 1 Central carbon metabolism data (LC-MS metabolomics): https://doi.org/10.21228/M87S00; Figures 2 and 3: Comparison of AA3 vs. AAGly data (LC-MS metabolomics): https://doi.org/10.21228/M8N274; Figure 4: Expression data of heme biosynthesis genes (qPCR): https://doi.org/10.5281/zenodo.19162793; Figure 5: ^13^C_2_-glycine labeling data (SIRM): https://doi.org/10.21228/M8CK39
• This study does not report original code.
• Any additional information required to reanalyze the data reported in this paper is available from the lead contact upon request.

## Acknowledgments

V.P.G., K.R., and S.M. are thankful for the access to the qPCR instrument from Dr. Seino Jongkees, at the VU Amsterdam. VPG and SM thank Daniëlle Gramsbergen and Dr. Johan van Heerden, at the VU Amsterdam, for contributing to the production of ^13^C yeast extract, and Prof. Dr. Ines Heiland for her valuable insights into isotope tracing dynamics. All authors thank Prof. Dr. Catherine Verfaillie and Dr. Sreya Ghosh for kindly providing the SBAD2-3X cells. This work was supported by the project RISK-HUNT3R: RISK assessment of chemicals integrating Human-centric Next generation Testing strategies promoting the 3Rs. RISK-HUNT3R has received funding from the European Union’s Horizon 2020 research and innovation program under grant agreement No 964537 and is part of the ASPIS cluster. This work reflects only the authors’ views, and the European Commission is not responsible for any use that may be made of the information it contains.

## Author contributions

V.P.G.: conceptualization, formal analysis, investigation, methodology, and writing – original draft. T.S.C.: formal analysis, software, writing - reviewing, and editing. K.R.: investigation, writing - reviewing, and editing. S.S.: formal analysis, writing - reviewing, and editing. P.J.: resources, writing - reviewing, and editing. J.C.V.: conceptualization and writing – original draft. S.M.: conceptualization, investigation, supervision, methodology, and writing – original draft.

## Declaration of interests

The authors declare no competing interests.

## Declaration of generative AI and AI-assisted technologies in the writing process

During the preparation of this work, V.P.G. used Grammarly to correct textual spelling and grammar. After using this tool, the authors reviewed and edited the content as needed and take full responsibility for the content of the publication.

## STAR★Methods

### Key resources table

**Table:** 

REAGENT or RESOURCE	SOURCE	IDENTIFIER
**Antibodies**		

HNF4 alpha	Merck Life Science	Cat#HPA004712; RRID:AB_1079075
CYP3A4	Abcam	Cat#AB124921; RRID:AB_2924823
Carboxylesterase 1 (CES1)	Thermo Fisher	Cat#PA5-19740; RRID:AB_10986106
Albumin	Dako	Cat#A0001; RRID:AB_2943648
Alpha 1 Anti-trypsin	Dako	Cat#20079116
Phosphoenolpyruvate carboxykinase (PEPCK)	Sigma-Aldrich	Cat#SAB5701540
Hoechst 33342	Thermo Fisher	Cat#11534886; AB_10626776
Alexa 647 donkey anti-rabbit IgG	Thermo Fisher	Cat#A32795; RRID:AB_2762835

**Chemicals, peptides, and recombinant proteins**		

DMSO for cell culture	Merck Life Science	Cat#D8418; CAS: 67-68-5
Collagen IV human placenta	Merck Life Science	Cat#27663; CAS: 9007-34-5
Human Recombinant Albumin	Merck Life Science	Cat#A9731
L-Ascorbic acid 2-phosphate	Merck Life Science	Cat#A8960; CAS: 1713265-25-8
Transferrin	Merck Life Science	Cat#T3705; CAS: 11096-37-0
Sodium selenite	Merck Life Science	Cat#S5261; CAS: 10102-18-8
Recombinant FGF2-G3 protein	Qkine	Cat#Qk053
Human Recombinant TGF-beta 1	StemCell Technologies	Cat#78067.1
Dexamethasone-Water Soluble	Merck Life Science	Cat#D-2915; CAS: 50-02-2
Hydrocortisone 21-hemisuccinate sodium salt	Merck Life Science	Cat#H2270; CAS: 125-04-2
Recombinant mouse Wnt3a protein	Abcam	Cat#ab81484
Human Recombinant BMP-4	StemCell Technologies	Cat#78211
Human Recombinant HGF	StemCell Technologies	Cat#78019.1
Human/Mouse Recombinant Activin A	StemCell Technologies	Cat#78001.1
Human Recombinant FGF-acidic	StemCell Technologies	Cat#78187.1
Y-27632 dihydrochloride, Rho kinase inhibitor	Abcam	Cat#Ab120129
Doxycycline hydrochloride	Merck Life Science	Cat#D3447; CAS: 10592-13-9
Oligomycin	Merck Life Science	Cat#O4876; CAS: 1404-19-9
FCCP	Merck Life Science	Cat#C2920; CAS: 370-86-5
Rotenone	Merck Life Science	Cat#R8875; CAS: 83-79-4
Antimycin A	Merck Life Science	Cat#A8674; CAS: 1397-94-0
HEPES	Merck Life Science	Cat#H4034; CAS: 7365-45-9
Glycine	Merck Life Science	Cat#G7126; CAS: 56-40-6
D-Glucose	Merck Life Science	Cat#G8270; CAS: 50-99-7
Sodium Pyruvate	Merck Life Science	Cat#P5280; CAS: 113-24-6
Sodium lactate	Merck Life Science	Cat#71718; CAS: 867-56-1
L-Glutamine	Merck Life Science	Cat#G8549; CAS: 56-85-9
Sodium chloride	Merck Life Science	Cat#31434; CAS: 7647-14-5
Potassium chloride	J.T. Baker	Cat#4001-01; CAS: 7447-40-7
Calcium chloride	Merck Life Science	Cat#1.02378; CAS: 10043-52-4
Magnesium sulfate heptahydrate	J.T. Baker	Cat#2505-07; CAS: 10034-99-8
Magnesium chloride hexahydrate	Merck Life Science	Cat#M2670; CAS: 7791-18-6
Sodium phosphate dibasic dihydrate	J.T. Baker	Cat#0326; CAS: 10028-24-7
Potassium phosphate monobasic	Merck Life Science	Cat#P5655; CAS: 7778-77-0
Sodium bicarbonate	VWR Chemicals	CAS: 144-55-8
L-Serine	Merck Life Science	Cat#S8407; CAS: 56-45-1
^13^C_2_-Glycine	CortecNet	Cat#CC1080P1; CAS: 20220-62-6
Triethanolamine HCl	Merck Life Science	Cat#T9534; CAS: 637-39-8
EDTA disodium salt dihydrate	PanReac AppliChem	Cat#A2937-0500; CAS: 6381-92-6
Phenazine methosulfate (PMS)	Merck Life Science	Cat#P9625; CAS: 299-11-6
Iodonitrotetrazolium chloride (INT)	Merck Life Science	Cat#I8377; CAS: 146-68-9
Sodium azide	Merck Life Science	Cat#S2002; CAS: 26628-22-8
Potassium acetate	VWR Chemicals	Cat#85507.290; CAS: 127-08-2
Glycogen from bovine liver	Merck Life Science	Cat#G0885; CAS: 9005-79-2
Glucose-6-phosphate Dehydrogenase from *Leuconostoc mesenteroides*	Merck Life Science	Cat#G8404; CAS: 9001-40-5
Hexokinase from *Saccharomyces cerevisiae*	Merck Life Science	Cat#H4502; CAS: 9001-51-8
NADP	PROZOMIX	Cat#PRO-NADP; CAS: 24292-60-2
Adenosine 5′-triphosphate disodium salt hydrate	Merck Life Science	Cat#A26209; CAS: 34369-07-8
Amyloglucosidase from *Aspergillus niger*	Merck Life Science	Cat#A7420; CAS: 9032-08-0
Triton X-100	Merck Life Science	Cat#T8787; CAS: 9036-19-5
Sodium Hydroxide	Merck Life Science	Cat#1.06498; CAS: 1310-73-2
Acetic acid	Merck Life Science	Cat#8.18755; CAS: 64-19-7
Ammonium Acetate	Merck Life Science	Cat#73594; CAS: 631-61-8
Ammonia solution 25%	Merck Life Science	Cat#1.05432.1011; CAS: 1336-21-6

**Critical commercial assays**		

RNeasy Mini Kit (50)	Qiagen	Cat#74104
iScript™ Reverse Transcription Supermix	Bio-Rad	Cat#1708841
Luna Universal qPCR Master Mix	BIOKE	Cat#NEB M3003E
Quant-iT™ PicoGreen™ dsDNA Assay Kit and dsDNA Reagent	Thermo Fisher	Cat#P7589
P450-Glo™ CYP3A4 Assay and Screening System	Promega	Cat#V9001
Soluble Collagen Quantification Assay Kit	Merck Life Science	Cat#CS0006

**Deposited data**		

RNA-Seq Data (TCGA liver hepatocellular carcinoma (LIHC) gene expression by RNAseq (polyA + IlluminaHiSeq))	The Cancer Genome Atlas (https://tcga.xenahubs.net) Public dataset	TCGA.LIHC.sampleMap/HiSeqV2
Figure 1: Cellular respiration data (Seahorse)	This paper	https://doi.org/10.5281/zenodo.19162793
Figure 1: Central carbon metabolism data (LC-MS metabolomics)	This paper	https://doi.org/10.21228/M87S00
Figures 2 and 3: Comparison of AA3 vs. AAGly data (LC-MS metabolomics)	This paper	https://doi.org/10.21228/M8N274
Figure 4: Expression data of heme biosynthesis genes (qPCR)	This paper	https://doi.org/10.5281/zenodo.19162793
Figure 5:^13^C_2_-glycine labeling data (SIRM)	This paper	https://doi.org/10.21228/M8CK39

**Experimental models: Cell lines**		

Human hepatoma HepG2	ECACC, UK Health Security Agency	N/A
iPSCs SBAD2-clone 1, STBCi321-A	Morrison et al., 2015	https://www.cellosaurus.org/CVCL_ZX54

**Oligonucleotides**		

Primers for *ALAS1, ALAD, FECH, UROS, CPOX, GAPDH*, see Table S3	OriGene Technologies	N/A

**Software and algorithms**		

Harmony 4.9 software	PerkinElmer	N/A
AriaDx Real-Time PCR V.2.1	Agilent Technologies	N/A
SCIEX OS V.3.1.6 software	SCIEX	N/A
GraphPad Prism 8.0.1	GraphPad	N/A

**Other**		

Fetal Bovine Serum, qualified, Brazil	Gibco	Cat#10270106
DMEM, low glucose, pyruvate	Gibco	Cat#31885023
MEM amino acids solution (50×)	Thermo Fisher	Cat#15040033
MEM non-essential amino acids solution (100×)	Thermo Fisher	Cat#12519059
Trypsin-EDTA solution	Merck Life Science	Cat#T4049
Geltrex™	Gibco	Cat#A14133-02
Versene Solution/EDTA	Thermo Fisher	Cat#15040033
Penicillin-Streptomycin	Merck Life Science	Cat#P4333
StemPro™ Accutase™ Cell Dissociation Reagent	Thermo Fisher	Cat#11599686
Albumin, Bovine Serum, Fraction V, Fatty Acid-Free, Nuclease- and Protease-Free	Merck Life Science	Cat#126609
DMEM, high glucose	Gibco	Cat#41965-039
Ham’s F-12 Nutrient Mix	Gibco	Cat#21765-029
Insulin	Gibco	Cat#A11382II
GlutaMAX	Gibco	Cat#35050-038
DMEM-Low glucose	Gibco	Cat#31885
MCDB 201 Medium	Merck Life Science	Cat#M6770
Insulin-Transferrin-Selenium (ITS -G) (100×)	Gibco	Cat#4140-045
Linoleic Acid-Albumin from bovine serum albumin	Merck Life Science	Cat#L9530
2-Mercaptoethanol (50 mM)	Gibco	Cat#31350010
Seahorse XF Base Medium without phenol red	Agilent Technologies	Cat#1003335-100
Dulbecco’s MEM (DMEM) w/L-Glutamine and Sodium Bicarbonate w/o Glucose, Serine, Glycine	CliniSciences	Cat#D9800-16
XF96 V3 PS Cell Culture Microplates	Agilent Technologies	Cat#101085-004
XFe96/XF Pro sensor cartridges	Agilent Technologies	Cat#103792-100

### Experimental model and study participant details

All cell models were maintained at 37 °C in a 5% CO_2_ humidified atmosphere. HepG2 and SBAD2-3X cell line authentication was conducted by Eurofins. Mycoplasma testing was performed regularly resulting negative while experiments were conducted.

#### HepG2 cell line

Human hepatoma HepG2 were purchased from the European Collection of Authenticated Cell Cultures (ECACC, UK Health Security Agency). Cells were cultured in DMEM media with low glucose (5.5 mM) and pyruvate (1 mM), 1% (v/v) penicillin/streptomycin, and 9% (v/v) fetal bovine serum (FBS). Cells were passaged with trypsin and seeded with a 45.000 cells/cm^2^ density. Forty-eight hours after seeding, cells (between passages 12 and 20) were used in experiments.

#### mHepG2

HepG2 were seeded with a density of 45.000 cells/cm^2^ and 5 days after, once 70–80% of well’s confluency was reached, media was supplemented with amino acids^4^ (AAHepG2 media: 16 mL of non-essential amino acids, 8 mL of essential amino acids solution, containing 100× and 50×, respectively, Minimum Essential Medium’s (MEM) amino acids concentration, except L-glutamine, and additional 20 g/L of glycine in 100 mL culture media, see Table S4). HepG2 were matured in AAHepG2 media for >20 days, before being used.

#### SBAD2-3X culture and differentiation into HLCs

iPSCs used in this study (SBAD2, STBCi321-A, https://www.cellosaurus.org/CVCL_ZX54) were generated using the CytoTune 2.0 (Thermo Fisher) Sendai viral reprogramming kit from normal dermal fibroblasts.^55^ iPSCs SBAD2-clone 1 (SBAD2) were genetically modified by using recombinase-mediated cassette exchange to obtain iPSCs (SBAD2-3X) overexpressing HNF1A, FOXA3, and PROXY1 through a doxycycline inducible cassette, as described elsewhere.^51^ SBAD2-3X were kindly provided by Prof. Dr. Catherine Verfaillie, KU Leuven.^51^ SBAD2-3X were cultured and differentiated into HLCs as detailed elsewhere.^18^ In short, for the differentiation into HLCs, SBAD2-3X were passaged with accutase and seeded at a density of 35.000 cells/cm^2^ in human embryonic stem cell qualified reduced growth factor basement membrane matrix (Geltrex) coated plates. Twenty-four hours after seeding, media was changed into LDM basal media. At day 12 of differentiation, high concentration of amino acids was added to the media (AA3 media). From day 14 onwards, glycine was added to AA3 media (AAGly media) with a final concentration of 20 g/L. Differentiation was carried out for >40 days. For media composition, see Tables S4, S5, and S6.

### Method details

#### Immunofluorescence

Quality control of HLCs differentiation was assessed by measuring six specific liver markers at days 22 and 44 of differentiation: CYP3A4, hepatocyte nuclear factor 4 (HNF4) alpha, carboxylesterase 1 (CES1), albumin, alpha 1 antitrypsin, and phosphoenolpyruvate carboxykinase (PEPCK) by immunofluorescence. Cell fixation and antibody staining were conducted as described elsewhere.^56^ The secondary antibody for all three primary antibodies was Alexa 647 donkey anti-rabbit IgG. The nucleus was stained with Hoechst 33342. The cells were imaged with the Operetta CLS High-Content Imager (PerkinElmer), using confocal imaging with a 20× water immersion objective. Image analysis was performed with the Harmony 4.9 software.

#### Mitochondrial respiration assay

Oxygen consumption rates (OCR) of the cell models (HepG2, mHepG2, day 22 and 44 HLCs) were tested using the Seahorse XFe96 Bioanalyzer from Agilent. The Mitostress assay was performed as previously described.^57^ All four cell models were seeded and differentiated onto Seahorse XF96 V3 PS Cell Culture Microplates. HLCs were plated on Geltrex coated XF96 V3 PS Cell Culture Microplates, while HepG2 were plated on collagen IV human placenta (10 μg/cm^2^) coated plates; for mHepG2 no coating was needed. Cells were pre-incubated for 2 h in a non-CO_2_ incubator at 37 °C with the Seahorse XF Base Medium (without phenol red), supplemented with 10 mM glucose, 5 mM HEPES, 2 mM sodium pyruvate, and 1 mM glutamine; volume per well was 180 μL. The Agilent XFe96 sensor cartridge was hydrated using SeahorseXF Calibrant 24 h prior to the assay. During the assay, OCR (pmol O_2_/min) was measured 3 times to determine basal respiration, and 3 times after injecting each mitochondrial modulator. ATP production, maximal respiration rate, and non-mitochondrial respiration were assessed by adding the following inhibitors: oligomycin (2 μM final concentration), carbonyl cyanide-*p*-trifluoromethoxyphenylhydrazone (FCCP, 2 μM), and rotenone/antimycin A (0.5 μM each). Results were normalized by cell count, measured with the nuclear staining dye Hoechst 33342 in the BioTek Cytation 1.

#### Glycogen determination

Glycogen levels were quantified from cell pellets after quenching and extraction of samples for metabolomics analysis. After extraction, pellets were dissolved in 100 μL of Mili-Q water and sonicated for 10 s; at this step, aliquots were separated to normalize the data by DNA amount. The remaining homogenate was used to determine glycogen, following a modified protocol.^58^ Shortly, 1 U of amyloglucosidase dissolved in 5 mM potassium acetate, 0.02% (m/v) sodium azide, and 0.02% (m/v) bovine serum albumin, was used for the degradation of glycogen found in each homogenate. After adding the enzyme, samples were incubated for 90 min at 37 °C in an Eppendorf Thermomixer Shaking Heater Block. Together with the samples, glycogen standards (40 nmol–0.3 nmol in 8 dilution steps) were also prepared. After glycogen degradation, samples and standards were centrifuged (15 min), and 60 μL of the supernatant was transferred to a transparent Greiner Bio-One 96-well Flat Bottom plate for glucose determination, following an established protocol.^59^ Shortly, the NADPH formed in the conversion of glucose to glucose-6-phosphate ultimately reduces the dye *p*-iodonitrotetrazolium violet (INT) to INTH, measured at 520 nm with a CLARIOstar Plus, BMG Labtech. Sixty microliters of the glucose assay reaction mixture^59^ was added to the 96-well plate and incubated for 20 min at room temperature. Water and glucose (60 μL) were included as blanks and controls, respectively.

#### Collagen determination

Collagen was measured using a Soluble Collagen Quantification Assay Kit (Merck Life Science, cat. CS0006). For the sample preparation, living cells in 6-well plates were washed with phosphate buffered saline solution (PBS) and detached using 0.5 mL of trypsin for 3 min. Harvested cells were collected in an Eppendorf containing 1 mL of media with 10% (v/v) FBS before 5 min centrifugation at 300 rcf. Cell pellets were washed with PBS, and after a second centrifugation, were resuspended in 0.5 mL of ice-cold 0.5 M acetic acid. Samples were sonicated and incubated overnight at 4 °C with gentle agitation. The next day, samples were centrifuged at 10000 rcf, 15 min, 4 °C. Four hundred microliters of the supernatant was transferred to a new Eppendorf and neutralized using 400 μL of 0.5 M NaCl. Collagen determination was performed following the provider’s instructions. The remaining pellet was collected for total DNA determination.

#### Heme biosynthesis gene expression

Gene expression of enzymes part of the heme biosynthesis pathway was determined by quantitative polymerase chain reaction (qPCR). Both HLCs and mHepG2 were cultured in 6-well plates and RNA was isolated using RNeasy Mini Kit (Qiagen, cat. 74104). Complementary DNA (cDNA) was obtained using the iScript Reverse Transcription Supermix (Bio-Rad, cat. 1708841). The final cDNA concentration for all tested genes in the qPCR reaction was 0.156 ng/μL. Luna Universal qPCR Master Mix (BIOKE, cat. NEB M3003E) was used for the qPCR reactions. The concentration and volume of reagents used followed the manufacturer’s specifications. Primers for 5-aminolevulinic acid synthase 1 (*ALAS1*), 5-aminolevulinic acid dehydratase (*ALAD*), ferrochelatase (*FECH*), uroporphyrinogen synthase (*UROS*), and coproporphyrinogen oxidase (*CPOX*) (see Table S3 for primer sequence) were first tested in AAGly mHepG2 as a standard curve, assuring an efficiency between 90 and 110%. The same fluorescence threshold (ΔR) was maintained across cell systems for each transcript (Table S3). Analysis was performed in AriaDx Real-Time PCR (qPCR) V.2.1 instrument from Agilent. Results were expressed as relative quantification of the gene of interest to the housekeeping gene, here *GAPDH*, following formula [1].

$$[1]\;\;\;\;\;\frac{2^{-C_{Tgene\;of\;intetest}}}{2^{-C_{T\;GAPDH}}}$$

To correct for inter-plate variations, a calibrator was used: cDNA of AAGly mHepG2 with a concentration of 0.156 ng/μL amplified with *GAPDH* (keeping the ΔR set at 20 for all measurements). When the gene of interest and *GAPDH* were amplified in different plates, before following formula [1], $2^{-C_{Tgene\;of\;interest}}$ was corrected by the average $2^{-C_{Taverage\;calibrator}}$ of the plate calibrator.

#### CYP3A4 activity determination

Glo assay (P450-Glo CYP3A4 Assay and Screening System, Promega, cat. V9001) is a luminescent method for measuring P450 CYP3A4 activity. CYP3A4 activity was measured in both mHepG2 and HLCs under the conditions of basal, AA3, and AAGly media, to assess glycine effect on CYP activity. This assay was also used to measure CYP3A4 activity in HepG2 after 72 h ALA exposure (0, 50, 150, 350, and 500 μM) in HepG2 culture media. Read-outs followed the indications of the manufacturer and were performed in 96-well plate format. The results were normalized by cell count, measured with a nuclear staining dye, Hoechst 33342, in the BioTek Cytation 1.

#### Carbon source exposure

Cell systems HepG2, mHepG2, day 22 HLCs, and day 44 HLCs were incubated with one of the following carbon sources: glucose, glutamine, lactate, pyruvate, glycine at 5 or 20 mM, prepared in Hank’s balanced salt solution (HBSS, see Table S7). Cells were seeded on 6-well plates (HLCs were cultured in Geltrex-coated plates) and incubated for 2 h with one carbon source. Cells were incubated with only HBSS as controls. After incubation, cell medium was discarded; the cells were washed with 0.9% (m/v) NaCl, and the intracellular content was immediately quenched with liquid nitrogen before sample preparation for metabolomics analysis. To compare metabolic changes across cell models exposed to different carbon sources, metabolite LC-MS intensities were normalized to levels in HBSS controls, for each cell system: $\frac{Metabolite\;intensity\;experimental\;condition/IS}{Metabolite\;intensity\;HBSS\;condition/IS};IS=internal\;standard$.

#### Glycine exposure along hepatic differentiation

HLCs and mHepG2 were cultured with and without 2% (m/v) glycine supplementation, and metabolic changes were assessed using metabolomics. HLCs were cultured for >40 days in either AA3 or AAGly media, added to the cells at day 14 of differentiation. mHepG2 cells were cultured from day 5 after seeding until day 20+ with AAHepG2 media with or without 2% (m/v) glycine in excess. Both conditions (here named AA3, low glycine, and AAGly, high glycine) were cultured in parallel along the same cellular differentiation, see media composition in Table S4. Once differentiation was finalized, cells were washed with 0.9% (m/v) NaCl, and the intracellular contents were immediately quenched with liquid nitrogen. Both mHepG2 and HLCs were seeded in 6-well plate format, coated with Geltrex for the HLCs, while mHepG2 did not require coating.

#### Cell proliferation with supplementation of 2% (m/v) glycine or 2% (m/v) glucose

HLCs and mHepG2 were cultured with 2% (m/v) glycine or 2% (m/v) glucose supplementing the AA3 media in a 96-well plate format, following the above-described timeline. Once differentiation was complete, number of cells per well was measured using a nuclear staining dye, Hoechst 33342, in the BioTek Cytation 1.

#### DNA determination

Total DNA amount was measured from the remaining pellet after extraction for metabolomics analysis. The kit used to quantify DNA was Quant-iT PicoGreen dsDNA Assay Kit and dsDNA Reagent (Thermo Fisher, cat. P7589). Manufacturer’s indications were followed.

#### ^13^C_2_-glycine tracing metabolomics

HLCs and mHepG2, cultured with low and high glycine concentrations (AA3 and AAGly), were incubated for 24 h at the end of the differentiation with ^13^C_2_-glycine (NH_2_^13^CH_2_COOH). Dulbecco’s MEM (DMEM) without glucose, serine, and glycine (CliniSciences, cat. D9800-16) was used for the experiment. Media was supplemented with 0.4 mM L-serine and 5.5 mM glucose to equal concentrations as culture conditions. Final concentrations of 1.5 mM (AA3) or 268 mM (AAGly) ^13^C_2_-glycine were added to the media, mimicking glycine amounts found in AA3 and AAGly media. Both mHepG2 and HLCs were seeded in 6-well plate format (coated with Geltrex for the HLCs). After 24 h ^13^C_2_-glycine incubation, cells were washed with 0.9% (m/v) NaCl, and the intracellular contents were immediately quenched with liquid nitrogen. Samples were extracted and run in the LC-MS system, and isotopologue correction was carried out using PICor, a Python-based tool.^60^ Isotopologue correction adjusted for the natural abundance of ^13^C, which would otherwise lead to inflated labeling signals and bias analysis.^60^ Data was represented as % unlabeled (M), one ^13^C (M+1) and two ^13^C (M+2) fractions per metabolite.

#### Sample preparation for LC-MS metabolomics

Intracellular samples, previously quenched with liquid nitrogen, were extracted by scraping the cells with 1.5 mL of 80% (v/v) MeOH/water, together with 50 μL of ^13^C internal standard (IS, yeast extract produced in-house). Samples were incubated at 4 °C for 30 min on the Eppendorf Thermomixer Shaking Heater Block, following a centrifugation step (15 min). Extracts were dried overnight in a vacuum centrifuge (CHRIST). After dissolving the lysates with 60% (v/v) acetonitrile/water, a final centrifugation was performed (5 min), and samples were analyzed immediately after, using LC-MS. Samples from the ^13^C_2_-glycine labeling experiment were extracted without IS.

#### LC-MS metabolome analysis

All solvents and solutions for metabolomics analyses were of LC-MS grade. Ultrapure water was obtained from a water purification system (Milli-Q EQ 7000, Merck Life Science). Separation was performed using a UHPLC (Agilent 1290 UHPLC), coupled to an SCIEX ZenoTOF 7600 system with a heated electrospray ionization source (ESI). Two types of chromatography were conducted to cover different metabolite panels: hydrophilic interaction liquid chromatography (HILIC), based on^69^ and reversed phase (RP), based on.^18^^,^^61^ The injection volume was 1 μL, except for the ^13^C_2_-glycine labeling experiment, where 3 μL were injected instead. Samples were maintained at 10 °C until the analysis. Sample order was randomized within each batch. List of metabolites acquired using both HILIC and RP, and their analytical properties can be found in Tables S1 and S2. For some of the identified metabolites, authentic standards were available. For the remaining metabolites, identification was performed using accurate mass (with a tolerance of <5 ppm) and retention time, reported in previous literature. Specifically, here putatively identified bile acids matched the elution order reported elsewhere with authentic standards.^62^

##### Central carbon metabolism intermediates

HILIC was used with a ZIC-pHILIC column (5 μM, polymeric, 150 × 2.1 mm) and a guard column ZIC-pHILIC (5 μm, polymeric, 20 × 2.1 mm). For the analysis, mobile phase A (water: 10 mM ammonium acetate (NH_4_Ac) and 0.04% (v/v) ammonium hydroxide) and mobile phase B (100% acetonitrile) were used at a flow rate of 0.2 mL/min. Gradient went from 90 to 25% of B in 16 min with a total run of 30 min (0–0.50 min: 90% B; 0.50–16 min: 25% B; 16–21 min: 25% B; 21.10–30 min: 90% B). The column temperature was maintained at 35 °C.

##### Bile acid profiling and heme intermediates

RP chromatography (Waters XBridge BEH C18 2.1 mm internal diameter x 100 mm long, 2.5 μm particle size and 130 Å pore size) with a guard column (Waters XBridge BEH C18 3.9 mm × 5 mm, 3.5 μm particle size, 130 Å pore size) was used, at 40 °C and 0.2 mL/min flow rate. A linear gradient was applied: 10 to 80% of B (0.1% (v/v) formic acid in acetonitrile) in 18 min (0–1 min: 10% B; 1–19 min: 80% B; 19–21 min: 80% B; 21.5–24 min: 10% B) with a total run of 24 min (A, 0.1% formic acid (v/v) in ultrapure water).

##### MS method

Acquisition was performed in TOF-MS full scan. The nebulizer and drying gas were maintained at a pressure of 40 psi, while the curtain gas and the CAD gas were at 35 and 8 psi, respectively. Source temperature was set at 400 °C. The accumulation time was 0.1 s. Capillary voltage was 5.5 kV for positive and 4.5 kV for negative ion mode; with a declustering potential of 50 and 80 V, respectively. Collision energy was set at 10 V for both ion modes. The data storage was performed in profile mode. Acquisition and data integration (AutoPeak algorithm) were done using SCIEX OS V.3.1.6 software.

### Quantification and statistical analysis

#### Analysis of metabolomics data

Univariate statistical analyses were performed using unpaired t-tests via GraphPad Prism 8.0.1, considering results significant when the *p*-value was lower than 0.05. Data were presented as mean ± SD for comparisons between conditions. Data cleaning and processing were conducted using R software (version 4.3). Unsupervised methods, including principal component analysis (PCA)^63^ with mean centering and transformation, were applied for data exploration. Supervised methods, specifically linear discriminant analysis (LDA),^64^ were employed to identify discriminative features between experimental conditions. For data processing, PCA, and LDA, various R packages were used, including reshape2, dplyr, stringr, MASS, readxl, tidyverse, ggord, and ggrepel. When investigating the effect of different carbon sources among *in vitro* models, the intensities of the added carbon sources (glutamine, lactate, pyruvate, glycine, and glucose) were excluded from the metabolomics data during PCA and LDA to avoid multicollinearity. Similarly, glycine intensities were removed during PCA when comparing AA3 and AAGly conditions. Metabolites with missing values in any condition were also removed. In LDA, “cell model” was specified as the categorical response variable, with the predictor variables being the metabolite abundances under each condition. The significance of each discriminant function in separating the cell models was quantified by the proportion of trace, facilitating the identification of conditions with the highest discriminative power.

#### Analysis of RNA-Seq data from TCGA-LIHC

RNA-Seq dataset ID: TCGA.LIHC.sampleMap/HiSeqV2 was obtained from the TCGA-liver hepatocellular carcinoma (LIHC) cohort using the UCSC Xena platform (https://xena.ucsc.edu/) as described elsewhere.^65^ The dataset contained the gene-level transcription estimates, as in log2(x+1) transformed RSEM normalized count. For this analysis, only individuals with solid tumor (01) and/or solid tissue control (11) conditions were selected, leading to 109 total biological samples. Differentially expressed genes (DEGs) were identified using the limma package^66^ (version 3.58.1) in R (version 4.3). A linear modeling approach was applied to analyze gene expression data, comparing both tissue types from patients in the LIHC cohort. Genes were considered significantly differentially expressed if they met the criteria of an adjusted *p*-value <0.01 (Benjamini-Hochberg method^67^) and an absolute log2 fold change (|logFC|) > 1. DEGs were grouped into upregulated and downregulated categories based on log2 fold-change values. Functional analysis was performed using the ClusterProfiler package^68^ (version 4.14), applying over-representation analysis (ORA) for GO biological processes^35^ and KEGG pathways^34^ in the *Homo sapiens* (hsa) database. Significant pathways were identified with thresholds of *p*-value <0.05 (Benjamini-Hochberg adjustment^67^) and q-value <0.2, and the top 15 results were visualized with dot plots.

## Supplemental information

Document S1. Figures S1–S5 and Tables S1–S7

Data S1. Contains data related to each Figure in the main article
