# Supplementary material for: Glycine: The missing link between carbohydrate and xenobiotic metabolism in the maturing human hepatocyte
Source: iScience. 2026 May 28;29(6):116113. doi: 10.1016/j.isci.2026.116113 (PMC13233763; doi:10.1016/j.isci.2026.116113)
Supplement: Document S1. Figures S1–S5 and Tables S1–S7 [file mmc1.pdf]

iScience, Volume *29*

Supplemental information

Glycine: The missing link between carbohydrate and xenobiotic metabolism in the maturing human hepatocyte

Victoria Pozo Garcia, Tuğçe S. Çobanoğlu, Konstantina Riga, Suraj Sharma, Paul Jennings, J. Chris Vos, and Sofia Moco

SUPPLEMENTARY FIGURES

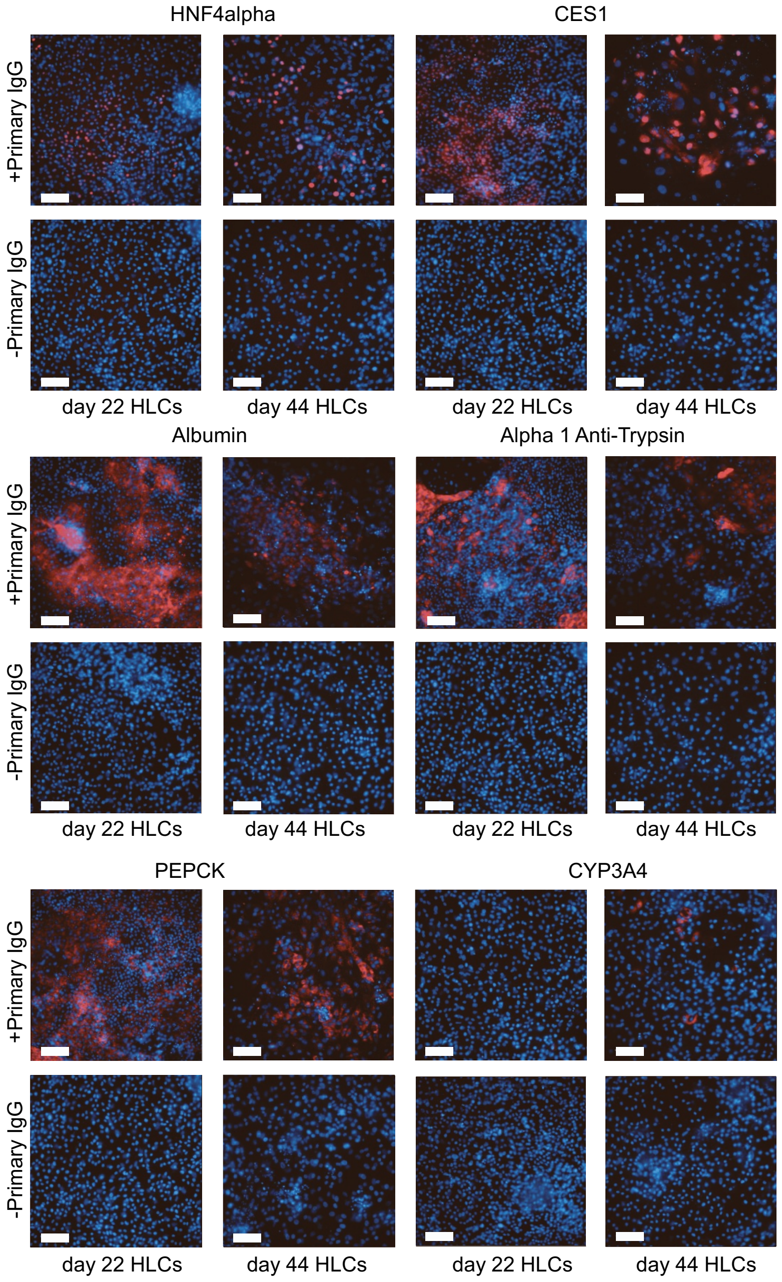

Figure S1. HNF4 alpha, CES1, albumin, alpha-1-antitrypsin, PEPCK, and CYP3A4 liver markers measured in HLCs day 22 and day 44 by immunofluorescence (extended information associated to Figure 1). Scale bar represents 100 µm. Imaged with the Operetta CLS High-Content Imager (PerkinElmer), using confocal imaging with a 20x water immersion objective. HNF4, hepatocyte nuclear factor 4; CES1, carboxylesterase 1; PEPCK, phosphoenolpyruvate carboxykinase (red). The nucleus (blue) was stained with Hoechst 33342.

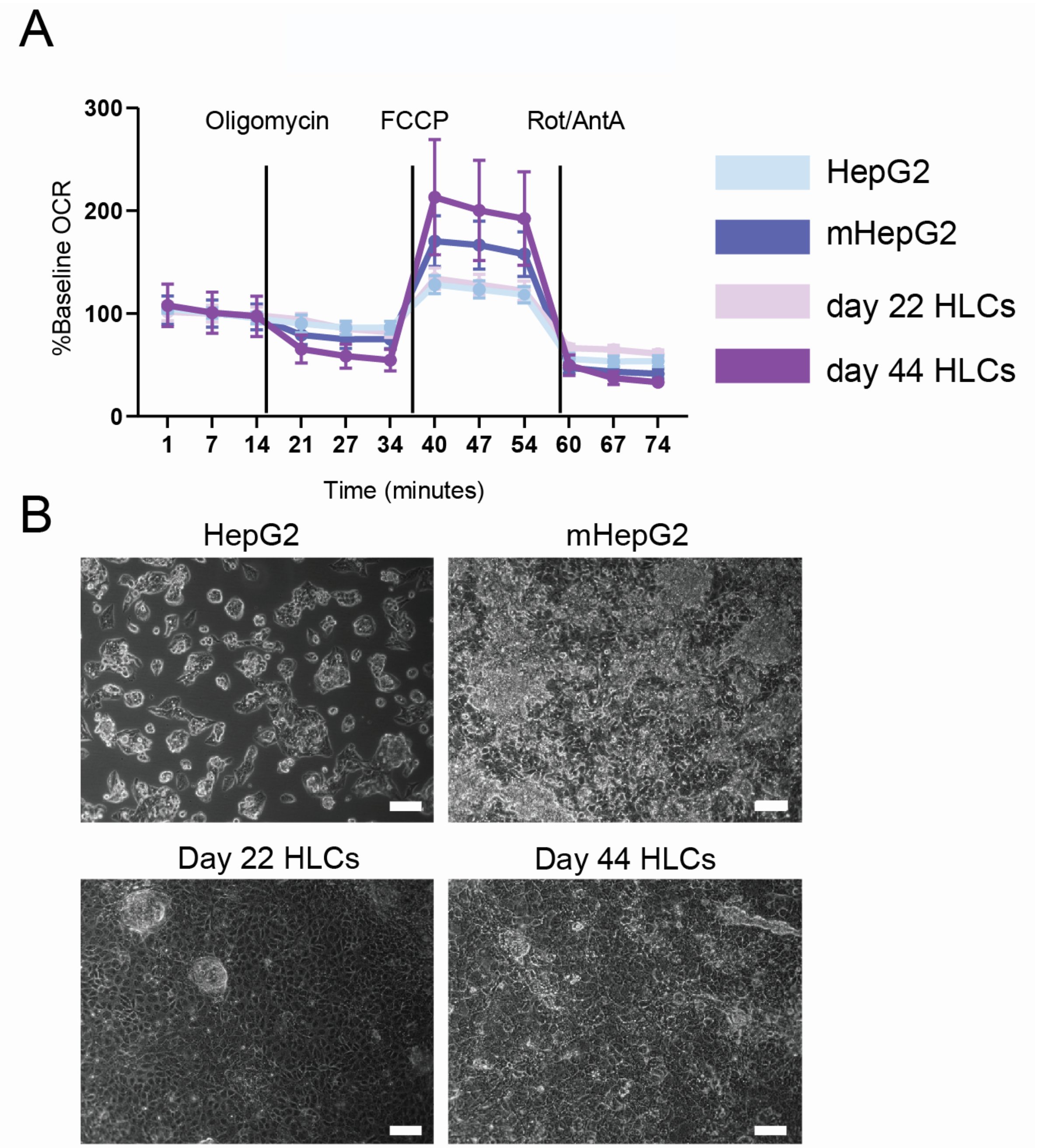

Figure S2. High concentration of amino acids increases mitochondrial spare capacity and oxidative metabolism in human liver cell models, (extended information associated to Figure 1). A. Mitostress assay profile using the Seahorse Bioanalyzer; light blue-HepG2, dark blue-mHepG2, pink-HLCs at day 22 of differentiation (day 22 HLCs), and purple-HLCs at day 44 of differentiation (day 44 HLCs). Data is represented as % to basal OCR (N=6-8) mean ± SD; **B**. Confocal images taken with a 10x objective of the liver *in vitro* models: HepG2, mHepG2, day 22, and day 44 HLCs. Scale bar represents 100 µm. FCCP: carbonyl cyanide-p-trifluoromethoxyphenylhydrazone, Rot/AntA: rotenone/antimycin A. N: separate wells of the same differentiation/cell batch independently processed. Source data are available for this figure.

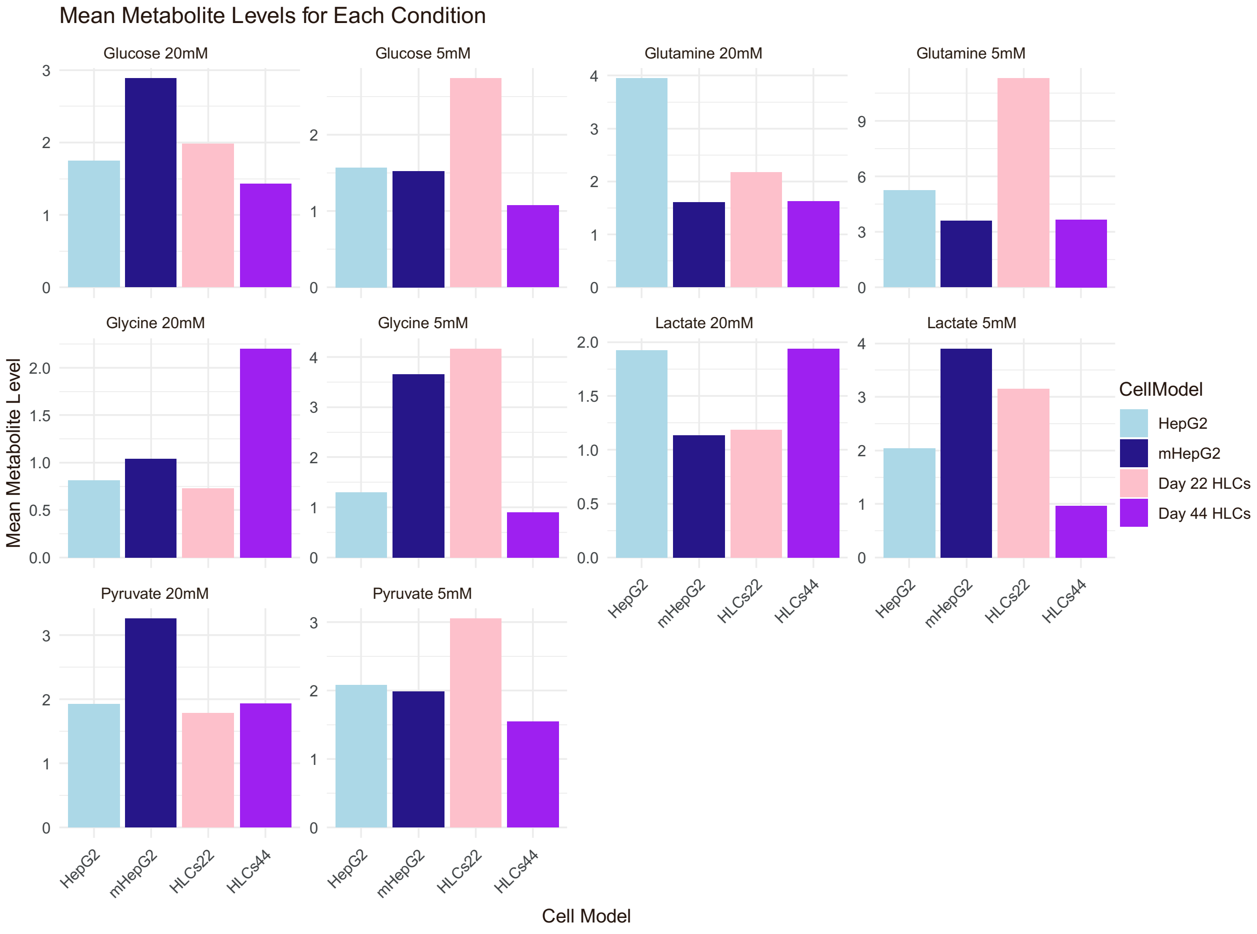

Figure S3. Mean metabolite levels obtained from LC-MS analysis of intracellular contents of HepG2, mHepG2, day 22 HLCs, and day 44 HLCs incubated for 2 h with different carbon source conditions: glucose, glycine, glutamine, pyruvate, and lactate, 5 and 20 mM (extended information associated to Figure 1). HepG2, light blue; mHepG2, dark blue; day 22 HLCs, pink; day 44 HLCs, purple (N=3). N: separate wells of the same differentiation/cell batch independently processed. Metabolite LC-MS intensities were normalized to levels in controls. Source data are available for this figure.

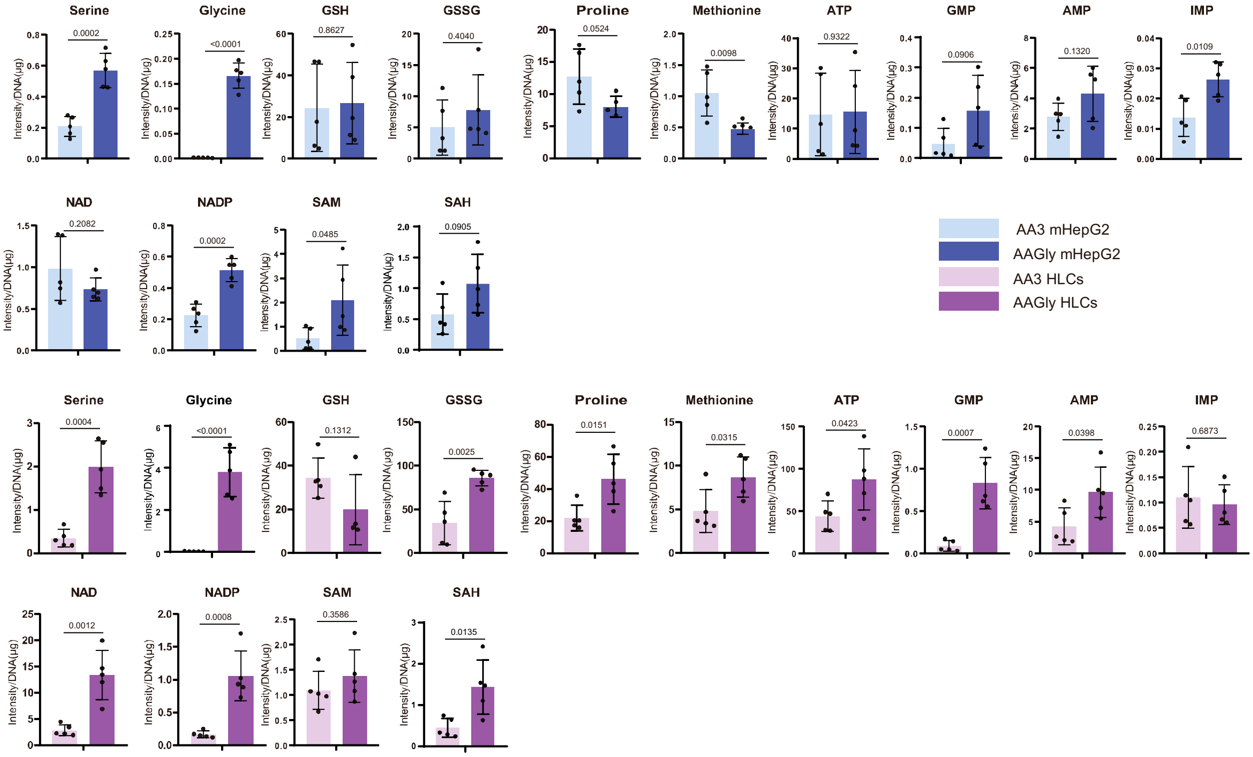

Figure S4. High glycine concentration increases 1C metabolism in HLCs but not in mHepG2 (extended information associated to Figure 2). Comparison of one-carbon metabolism intermediates (LC-MS intensities normalized to total DNA (μg)), in the two tested liver cell models, with and without high glycine conditions. Statistical differences were assessed using a t-test, considering significance when p-value<0.05. Individual replicates are represented as dots and error bars ± SD. AA3 mHepG2, light blue; AAGly mHepG2, dark blue; AA3 HLCs, pink; AAGly HLCs, purple (N=5). N: separate wells of the same differentiation batch independently processed. Source data are available for this figure.

Metabolite names in **Table S1.**

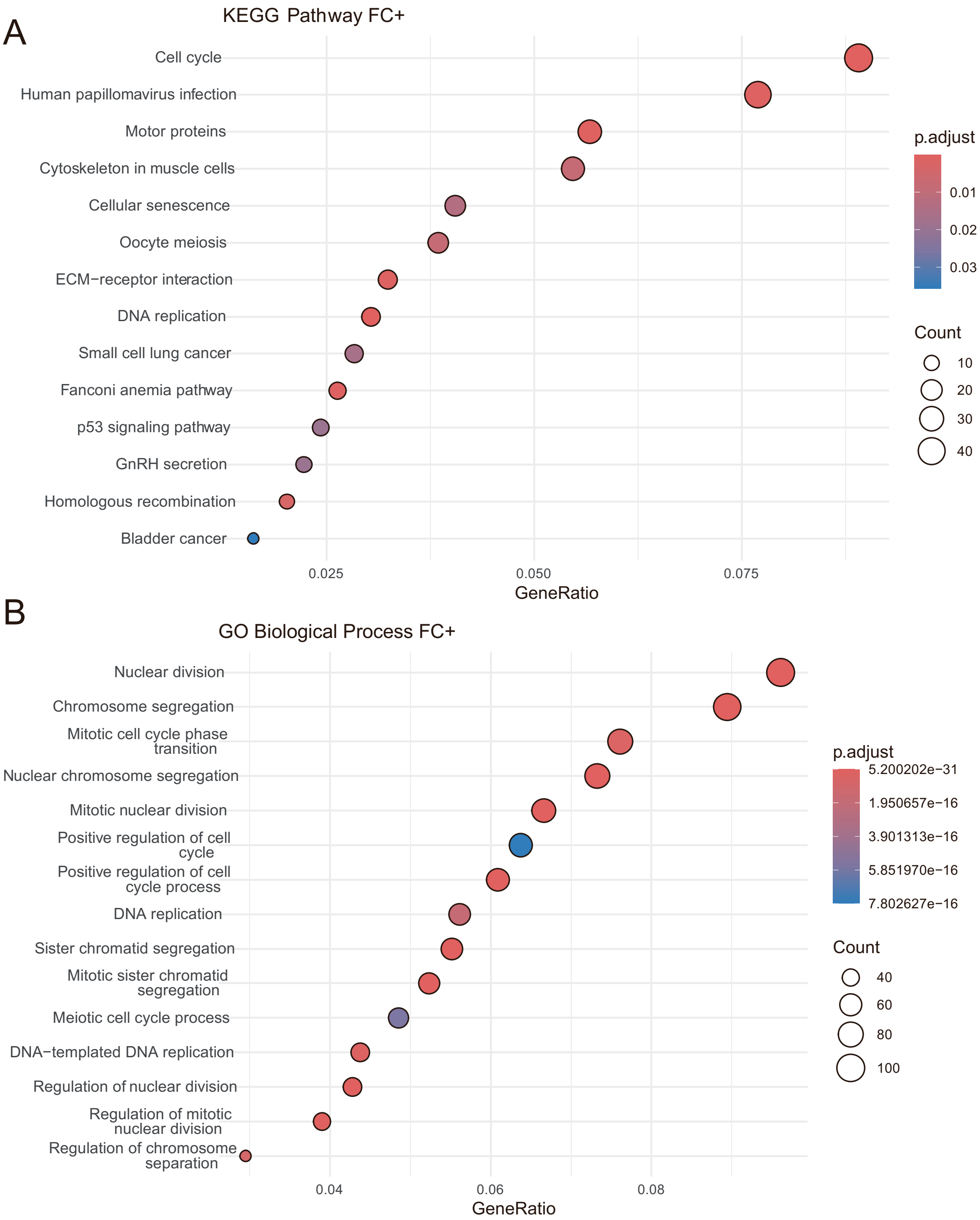

Figure S5. TCGA LIHC cohort (extended information associated to Figure 6). A. KEGG pathway enrichment analysis of significantly upregulated genes in cancer tissue compared to control liver tissue; **B.** GO biological process enrichment analysis of significantly upregulated genes in cancer tissue compared to control liver tissue.

SUPPLEMENTARY TABLES

Table S1, related to STAR Methods. Targeted list of metabolites analyzed by HILIC-based LC-MS; MF: molecular formula; m/z: mass over charge; RT [min]: retention time in minutes; ppm: mass error in ppm; ASC: authentic standard confirmation; IS assigned: ^{13}C internal standard assignment for intensity normalization (in-house yeast extract); [U-^{13}C]: ^{13}C uniformly labeled.

Metabolite	MF	Theoretical m/z	Ion polarity	RT (min)	ppm	ASC	IS assigned
Aminobutyric acid	$C_4H_9NO_2$	102.056	negative	9.4	0.1	yes	[U-^{13}C]Aminobutyric acid
(Iso)leucine	$C_6H_{13}NO_2$	132.102	positive	7.0	0	yes	[U-^{13}C]Glutamine
Adenosine 5′-diphosphate (ADP)	$C_{10}H_{15}N_5O_{10}P_2$	428.037	positive	10.8	-0.6	yes	[U-^{13}C]AMP
Alanine	$C_3H_7NO_2$	90.055	positive	9.1	0.2	yes	[U-^{13}C]Glutamine
Adenosine 5′-monophosphate (AMP)	$C_{10}H_{14}N_5O_7P$	348.070	positive	9.5	0	yes	[U-^{13}C]AMP
Arginine	$C_6H_{14}N_4O_2$	175.119	positive	14.8	-0.1	yes	[U-^{13}C]Arginine
Asparagine	$C_4H_8N_2O_3$	133.061	positive	9.3	-0.2	yes	[U-^{13}C]Glutamine
Aspartate	$C_4H_7NO_4$	134.045	positive	10.0	0.3	yes	[U-^{13}C]Glutamine
Adenosine 5′-triphosphate (ATP)	$C_{10}H_{16}N_5O_{13}P_3$	508.003	positive	11.1	0	yes	[U-^{13}C]AMP
Cytidine 5′-diphosphocholine (CDP)	$C_9H_{15}N_3O_{11}P_2$	404.025	positive	11.4	0	no	[U-^{13}C]AMP
Citrulline	$C_6H_{13}N_3O_3$	176.103	positive	9.7	0.4	yes	[U-^{13}C]Glutamine
Cytidine 5′-monophosphate (CMP)	$C_9H_{14}N_3O_8P$	324.059	positive	10.2	-0.1	no	[U-^{13}C]AMP
Creatine	$C_4H_9N_3O_2$	132.077	positive	8.9	0.5	yes	[U-^{13}C]Glutamine
Creatinine	$C_4H_7N_3O$	114.066	positive	5.7	0	yes	[U-^{13}C]Glutamine
Cytidine 5′-triphosphate (CTP)	$C_9H_{16}N_3O_{14}P_3$	483.992	positive	12.2	-0.1	no	[U-^{13}C]AMP
Citrate	$C_6H_8O_7$	191.020	negative	11.5	-0.9	yes	[U-^{13}C]Glutamate
Flavin adenine dinucleotide (FAD^+)	$C_{27}H_{33}N_9O_{15}P_2$	784.150	negative	8.5	-0.3	yes	[U-^{13}C]Glutamate
Fructose-1,6-bisphosphate (F1,6BP)	$C_6H_{14}O_{12}P_2$	338.989	negative	12.9	0.3	yes	[U-^{13}C]Glucose-phosphate
Fumarate	$C_4H_4O_4$	115.004	negative	10.5	-2.6	yes	[U-^{13}C]Glutamate
Glucose-6-phosphate (G6P)	$C_6H_{13}O_9P$	259.022	negative	10.7	-0.1	yes	[U-^{13}C]Glucose-6-phosphate
Guanosine 5′-diphosphate (GDP)	$C_{10}H_{15}N_5O_{11}P_2$	442.017	negative	12.0	-0.7	yes	[U-^{13}C]Glucose-phosphate

Glycerol-phosphate	$C_3H_9O_6P$	171.006	negative	9.9	-2.4	no	[U-^{13}C]Glutamate
Guanosine 5′-monophosphate (GMP)	$C_{10}H_{14}N_5O_8P$	362.051	negative	10.7	-0.4	yes	[U-^{13}C]UDP-Glucose
Lactate	$C_3H_6O_3$	89.024	negative	7.3	0.1	yes	[U-^{13}C]Glutamate
Malate	$C_4H_6O_5$	133.014	negative	10.5	-0.3	yes	[U-^{13}C]Glutamate
N-Acetyl aspartate	$C_6H_9NO_5$	174.041	negative	9.8	-0.5	no	[U-^{13}C]UDP-Glucose
Succinyl-CoA	$C_{25}H_{40}N_7O_{19}P_3S$	868.139	positive	9.8	2.5	no	-
Pyruvate	$C_3H_4O_3$	87.009	negative	10.1	-0.3	yes	[U-^{13}C]Glutamate
Xylulose-phosphate	$C_5H_{11}O_8P$	229.012	negative	10.2	-0.2	yes	[U-^{13}C]Glucose-phosphate
Cystathionine	$C_7H_{14}N_2O_4S$	223.075	positive	10.4	0	no	[U-^{13}C]AMP
Glutamate	$C_5H_9NO_4$	148.060	positive	9.6	0.1	yes	[U-^{13}C]Glutamate
Glutamine	$C_5H_{10}N_2O_3$	147.076	positive	9.2	0	yes	[U-^{13}C]Glutamine
Glycine	$C_2H_5NO_2$	76.039	positive	9.5	0	yes	[U-^{13}C]Glutamine
Glutathione (GSH)	$C_{10}H_{17}N_3O_6S$	308.091	positive	9.4	0	yes	[U-^{13}C]AMP
Glutathione oxidized (GSSG)	$C_{20}H_{32}N_6O_{12}S_2$	613.159	positive	11.2	0	yes	[U-^{13}C]AMP
Guanosine 5′-triphosphate (GTP)	$C_{10}H_{16}N_5O_{14}P_3$	523.998	positive	12.8	0	no	[U-^{13}C]AMP
Histidine	$C_6H_9N_3O_2$	156.077	positive	9.1	-1.8	yes	[U-^{13}C]Glutamine
Inosine 5′-monophosphate disodium (IMP)	$C_{10}H_{13}N_4O_8P$	349.054	positive	10.4	0.5	yes	[U-^{13}C]AMP
Kynurenine	$C_{10}H_{12}N_2O_3$	209.092	positive	7.1	-0.2	no	[U-^{13}C]Glutamine
Lysine	$C_6H_{14}N_2O_2$	147.113	positive	14.3	0.4	yes	[U-^{13}C]Glutamine
Methionine	$C_5H_{11}NO_2S$	150.058	positive	7.5	1.1	yes	[U-^{13}C]Glutamine
Nicotinamide adenine dinucleotide (NAD^+)	$C_{21}H_{27}N_7O_{14}P_2$	664.116	positive	9.3	0	yes	[U-^{13}C]NAD
Nicotinamide adenine dinucleotide phosphate ($NADP^+$)	$C_{21}H_{28}N_7O_{17}P_3$	744.083	positive	11.1	0.1	yes	[U-^{13}C]NAD
Ornithine	$C_5H_{12}N_2O_2$	133.097	positive	13.3	0.3	no	[U-^{13}C]Glutamine
Pantothenic acid	$C_9H_{17}NO_5$	220.118	positive	6.9	0.5	yes	[U-^{13}C]Glutamine
Phenylalanine	$C_9H_{11}NO_2$	166.086	positive	6.8	-0.9	yes	[U-^{13}C]Glutamine
Phosphocholine	$C_5H_{14}NO_4P$	184.073	positive	9.7	-0.2	no	[U-^{13}C]Glutamine
Phosphocreatine	$C_4H_{10}N_3O_5P$	212.043	positive	10.1	-0.5	no	[U-^{13}C]Glutamine
Phosphoserine	$C_3H_8NO_6P$	186.016	positive	10.8	1.7	no	[U-^{13}C]Glutamine
Proline	$C_5H_9NO_2$	116.071	positive	7.8	0.5	yes	[U-^{13}C]Glutamine
S-(5′-Adenosyl)-L-methionine iodide (SAM)	$C_{15}H_{22}N_6O_5S$	399.145	positive	10.4	0.9	yes	[U-^{13}C]AMP
Serine	$C_3H_7NO_3$	106.050	positive	9.6	0.7	yes	[U-^{13}C]Glutamine

S-Adenosyl-L-homocysteine (SAH)	$C_{14}H_{20}N_6O_5S$	385.129	positive	8.6	0.7	yes	[U-^{13}C]Glutamine
Taurine	$C_2H_7NO_3S$	126.022	positive	9.0	-2	yes	[U-^{13}C]Glutamine
Threonine	$C_4H_9NO_3$	120.066	positive	9.0	-0.6	yes	[U-^{13}C]Glutamine
Tyrosine	$C_9H_{11}NO_3$	182.081	positive	8.3	-0.2	yes	[U-^{13}C]Glutamine
Uridine 5'-diphosphate (UDP)	$C_9H_{14}N_2O_{12}P_2$	405.009	positive	10.7	0	no	[U-^{13}C]AMP
Uridine 5'-monophosphate (UMP)	$C_9H_{13}N_2O_9P$	325.043	positive	10.2	0	yes	[U-^{13}C]AMP
Uridine 5'-triophosphate (UTP)	$C_9H_{15}N_2O_{15}P_3$	484.976	positive	11.9	0	no	[U-^{13}C]AMP
Valine	$C_5H_{11}NO_2$	118.086	positive	7.8	-0.5	yes	[U-^{13}C]Glutamine
Sedoheptulose-7-phosphate	$C_7H_{15}O_{10}P$	289.033	negative	10.5	-3.1	yes	[U-^{13}C]Sedoheptulose-7-phosphate
Succinate	$C_4H_6O_4$	117.019	negative	10.1	0	yes	[U-^{13}C]Glutamate
UDP-Glucose	$C_{15}H_{24}N_2O_{17}P_2$	565.048	negative	10.5	-3.1	yes	[U-^{13}C]UDP-Glucose
UDP-*N*-acetylglucosamine	$C_{17}H_{27}N_3O_{17}P_2$	606.074	negative	10.2	-2.0	yes	[U-^{13}C]UDP-N-acetylglucosamine

Table S2, related to STAR Methods. Targeted list of heme biosynthesis metabolites and bile acids analyzed using RP-based chromatography LC-MS. MF: molecular formula; m/z: mass over charge; RT [min]: retention time in minutes; ppm: mass error in ppm; ASC: authentic standard confirmation; IS assigned: ^{13}C internal standard assignment for intensity normalization; [U-^{13}C]: ^{13}C uniformly labeled.

**Metabolite**	**MF**	**Theoretical m/z**	**Ion polarity**	**RT (min)**	**ppm**	**ASC**	**IS assigned**
5-Aminolevulinic acid	$C_5H_9NO_3$	132.066	positive	1.7	-0.3	yes	[U-^{13}C]Glutamine
Porphobilinogen	$C_{10}H_{14}N_2O_4$	227.103 (210.076*)	positive	2.1	1	yes	[U-^{13}C]Glutamine
CA (cholic acid)	$C_{24}H_{40}O_5$	407.280	negative	13.3	0	no	[U-^{13}C]Aminobutyric acid
GCA (glycocholic acid)	$C_{26}H_{43}NO_6$	464.302	negative	11.7	-2.6	no	[U-^{13}C]Aminobutyric acid
GDCA (glycodeoxycholic acid)	$C_{26}H_{43}NO_5$	448.307	negative	13.6	-0.2	no	[U-^{13}C]Aminobutyric acid
TCA (taurocholic acid)	$C_{26}H_{45}NO_7S$	514.284	negative	10.3	-0.5	no	[U-^{13}C]Aminobutyric acid
TCDCA (taurochenodeoxycholic acid)	$C_{26}H_{45}NO_6S$	498.289	negative	11.8	0.1	no	[U-^{13}C]Aminobutyric acid
TDCA (taurodeoxycholic acid)	$C_{26}H_{45}NO_6S$	498.289	negative	12.2	0.1	no	[U-^{13}C]Aminobutyric acid

*****Detected at 210.076 m/z (also authentic standard)

Table S3, related to STAR Methods. Primer sequences and threshold of fluorescence values (ΔR) set to quantify each gene: *ALAS1,* 5-aminolevulinic acid synthase 1; *ALAD*, 5-aminolevulinic acid dehydratase; *FECH*, ferrochelatase; *UROS*, uroporphyrinogen III synthase; *CPOX*, coproporphyrinogen oxidase; *GAPDH*, glyceraldehyde 3-phosphate dehydrogenase.

Gene	Sequence	ΔR
ALAS1	Fw. GATGTCAGCCACCTCAGAGAAC	31
	Rv. CATCCACGAAGGTGATTGCTCC	
ALAD	Fw. GACATACAGCCTATCACCAGCC	20
	Rv. CGCCAAAGATCAAGACACAGCG	
FECH	Fw. TCTTCTTGGACCGAGACCTCATG	22.5
	Rv. TCCAATCCTGCGGTACTGCTCT	
UROS	Fw. CAGACAGTTGCACACCCAGGAA	13
	Rv. GTGCTTGAGACTGTATGTGAGGC	
CPOX	Fw. TGAAGGAGGCTTGTGACCAGCA	18
	Rv. AGCGAAACACCTCCTCCTTGGA	
GAPDH	Fw. GTC TCC TCT GAC TTC AAC AGC G	20
	Rv. ACC ACC CTG TTG CTG TTG CTG TAG CCA A	

Table S4, related to STAR Methods. Final amino acid concentration and carbon sources in LDM, AA3 HLCs, AAGly HLCs, HepG2, AA3 mHepG2 and AAGly mHepG2, in mM.

	LDM	AA3HLCs	AAGlyHLCs	HepG2	AA3mHepG2	AAGlymHepG2
Glycine	0.2	1.5	267.9	0.4	1.61	268.0
L-Arginine hydrochloride	0.23	2.12	2.12	0.40	2.25	2.25
L-Cystine 2HCl	0.12	0.42	0.42	0.20	0.48	0.48
L-Glutamine	2.69	2.17	2.17	4.00	3.23	3.23
L-Histidine hydrochloride-H2O	0.11	0.74	0.74	0.20	0.81	0.81
L-Isoleucine	0.46	1.66	1.66	0.80	1.94	1.94
L-Leucine	0.46	1.66	1.66	0.80	1.94	1.94
L-Lysine hydrochloride	0.46	1.65	1.65	0.80	1.92	1.92
L-Methionine	0.12	0.42	0.42	0.20	0.49	0.49
L-Phenylalanine	0.23	0.83	0.83	0.40	0.97	0.97
L-Serine	0.23	1.48	1.48	0.40	1.61	1.61
L-Threonine	0.46	1.66	1.66	0.80	1.93	1.93
L-Tryptophan	0.04	0.20	0.20	0.08	0.22	0.22
L-Tyrosine disodium salt dihydrate	0.23	0.83	0.83	0.40	0.96	0.96
L-Valine	0.46	1.66	1.66	0.80	1.94	1.94
L-Alanine	0.00	1.30	1.30	0.00	1.30	1.30
L-Asparagine	0.00	1.30	1.30	0.00	1.30	1.30
L-Aspartic acid	0.00	1.30	1.30	0.00	1.30	1.30
L-Glutamic acid	0.00	1.30	1.30	0.00	1.30	1.30

L-Proline	0.00	1.30	1.30	0.00	1.30	1.30
D-Glucose	6.38	5.77	5.77	5.56	4.48	4.48
Pyruvate	0.77	0.62	0.62	1.00	0.81	0.81

Table S5, related to STAR Methods. Composition of LDM media.

Component	Final Concentration
DMEM-Low glucose	57%
MCDB dissolved in 1 L H_20 pH-7.1	40%
Penicillin-Streptomycin	/
L-Ascorbic acid in H_2O	0.1 mM
Insulin-Transferrin-Selenium (ITS -G) (100X)	/
Linoleic Acid-Albumin from bovine serum albumin	/
2-Mercaptoethanol (50 mM)	0.05 mM
Dexamethasone in H_2O	1 µM
Hydrocortisone hemisuccinate in H_2O	10 µM

Table S6, related to STAR Methods. Final concentration of cytokines in HLCs differentiation.

Cytokine	Final concentration in media
Recombinant mouse Wnt3a protein	0.05 µg/mL
Human Recombinant BMP-4	0.05 µg/mL
Human Recombinant HGF	0.02 µg/mL
Human/Mouse Recombinant Activin A	0.05 µg/mL
Human Recombinant FGF-acidic	0.02 µg/mL
Y-27632 dihydrochloride, Rho kinase inhibitor	10 µM
Doxycycline hydrochloride	5 µg/mL

Table S7, related to STAR Methods. Final concentration (mM) of Hank's balanced salt solution (HBSS), adjusted to pH 7.4.

Salt	Concentration in ultrapure water
Sodium Chloride	140
Potassium Chloride	5
Calcium Chloride	1
Magnesium Sulfate Heptahydrate	0.4
Magnesium Chloride Hexahydrate	0.5
Sodium Phosphate Dibasic Dihydrate	0.3
Potassium Phosphate Monobasic	0.4
Sodium Bicarbonate	4
